# Dendrons and Dendritic Terpolymers: Synthesis, Characterization and Self-Assembly Comparison

**DOI:** 10.3390/molecules25246030

**Published:** 2020-12-19

**Authors:** Sofia Rangou, Dimitrios Moschovas, Ioannis Moutsios, Gkreti-Maria Manesi, Konstantina Tsitoni, Polina V. Bovsunovskaya, Dimitri A. Ivanov, Edwin L. Thomas, Apostolos Avgeropoulos

**Affiliations:** 1 Department of Materials Science Engineering, University of Ioannina, University Campus-Dourouti, 45110 Ioannina, Greece; sofia.rangou@hzg.de (S.R.); dmoschov@uoi.gr (D.M.); imoutsios@uoi.gr (I.M.); gretimanesi@uoi.gr (G.-M.M.); k.tsitoni@uoi.gr (K.T.); 2 Helmholtz-Zentrum Geesthacht, Institute of Polymer Research, Max-Plank-Str. 1, 21502 Geesthacht, Germany; 3 Faculty of Chemistry, Lomonosov Moscow State University (MSU), GSP-1, 1-3 Leninskiye Gory, 119991 Moscow, Russia; bovspolina@yandex.ru (P.V.B.); dimitri.ivanov@uha.fr (D.A.I.); 4 Institute of Problems of Chemical Physics, Russian Academy of Sciences, Chernogolovka, 142432 Moscow, Russia; 5 Institut de Sciences des Matériaux de Mulhouse–IS2M, CNRS UMR7361, 15 Jean Starcky, 68057 Mulhouse, France; 6 Department of Materials Science and Engineering, Texas A&M University, College Station, TX 77843-3003, USA; elt@exchange.tamu.edu

**Keywords:** miktoarm stars, terpolymers, dendrons, dendritic terpolymers, characterization in solution, microphase separation, TEM, SAXS

## Abstract

To the best of our knowledge, this is the very first time that a thorough study of the synthetic procedures, molecular and thermal characterization, followed by structure/properties relationship for symmetric and non-symmetric second generation (2-G) dendritic terpolymers is reported. Actually, the synthesis of the non-symmetric materials is reported for the first time in the literature. Anionic polymerization enables the synthesis of well-defined polymers that, despite the architecture complexity, absolute control over the average molecular weight, as well as block composition, is achieved. The dendritic type macromolecular architecture affects the microphase separation, because different morphologies are obtained, which do not exhibit long range order, and various defects or dislocations are evident attributed to the increased number of junction points of the final material despite the satisfactory thermal annealing at temperatures above the highest glass transition temperature of all blocks. For comparison reasons, the initial dendrons (miktoarm star terpolymer precursors) which are connected to each other in order to synthesize the final dendritic terpolymers are characterized in solution and in bulk and their self-assembly is also studied. A major conclusion is that specific structures are adopted which depend on the type of the core connection between the ligand and the active sites of the dendrons.

## 1. Introduction

Living polymerization techniques provide the opportunity to synthesize polymers with predictable molecular characteristics and monodisperse distributions. The aforementioned methodologies, due to the living nature of the propagating chain-ends, allow the synthesis of well-defined non-linear polymers, with precise molecular architectures such as star- [1,2,3,4,5,6,7,8,9,10,11,12,13], H- [14,15,16,17,18,19,20,21,22,23], and dendritic-shaped materials [24,25,26,27,28,29,30], respectively. For the synthesis of dendritic polymers, anionic [31,32,33,34], click-chemistry [35] and organometal-catalyzed [36] techniques have already been employed and are reported in the literature. Due to their distinctive structures, dendritic polymers and/or dendrimers exhibit unique properties, rendering them suitable to be widely used in various applications [37,38], such as: drug delivery [39,40,41,42], catalytic systems [43,44], membranes [45], bioimaging [46,47], etc.

The combination of anionic polymerization and chlorosilane chemistry [23,48,49,50] may lead to the successful synthesis of both symmetric and asymmetric dendritic terpolymers consisting of three chemically different polymeric chains.

The definition of symmetric and asymmetric dendritic terpolymers is used, in order to point out the difference in the species of polymeric chains which constitute the first generation of the complex final architecture. In the case of symmetric dendritic terpolymers, the same type of segments is combined into the core (first generation), while two types of chemically different chains configure the outer generation as already reported in the literature by the Avgeropoulos group [34]. In this manuscript, it is the first time that asymmetric dendritic terpolymers are synthesized. The inner core is an A_2_B or A_3_B type miktoarm star copolymer and the outer generation consists of the remaining segments of the three or four initial dendrons, which when linked lead to the synthesis of the desired final material.

A very thorough and analytical study of the synthesis, molecular characterization and theoretical results of second generation dendritic homopolymers exhibiting compositional, as well as molecular homogeneity, and consisting of either poly(butadiene) (PB_1,4_) (with ~92% 1,4-microstructure) or poly(isoprene) (PI_3,4_) (with increased 3,4-microstructure, ~60%) have been reported by the Avgeropoulos group [32]. The same group has studied relevant symmetric copolymers [33] and terpolymers [34] for the first time. In the case of the dendritic copolymers comprising two chemically immiscible polydiene blocks (PB_1,4_ and PI_3,4_), well-organized hexagonally close-packed cylinders of PI in the PB matrix for different molecular characteristics of the two blocks were observed. This result verified the microphase separation potential of the two polydienes with specific microstructures as already mentioned in the literature by Avgeropoulos et al. for simpler linear triblock terpolymers of the PB_1,4_-*b*-PS-*b*-PI_3,4_ and PS-*b*-PB_1,4_-*b*-PI_3,4_ sequences [51]. 

Symmetric dendritic terpolymers have been mentioned in the literature, which were synthesized by using click-chemistry and ATRP techniques [52]. In addition, Hirao et al. synthesized a series of symmetric and asymmetric dendritic homopolymers of methyl methacrylate up to the seventh generation, by precisely controlling both the architecture and the length of the polymer chain [53,54].

The bulk phase separation of dendritic polymers, consisting of non-linear triblock terpolymers, is strongly dependent on the non-linear triblock precursors, their total degree of polymerization *N*, the three different Flory–Huggins interaction parameters: *χ_ΑΒ_, χ_BC_, χ_CA_*, the volume fraction of each segment: *φ_Α_, φ_Β_* (*φ_C_* = *1* − *φ_Α_* − *φ_Β_*) and the total number of junction points. Dendritic polymers rarely underlie to internal chain entanglements, due to intermolecular and intramolecular interactions, contrary to corresponding linear polymers exhibiting similar total number-average molecular weight [55].

Self-assembly on such complex terpolymers remains quite unexplored up to date. This fact may be attributed to the complexity of the architecture in these systems and the major difficulties in their synthesis. Asymmetric dendritic-like linear diblock copolymers or dendrimers were synthesized [56,57,58] and the subsequent morphological characterization indicated that the preliminary hexagonal topology was not evident after increasing the number of generations, moreover, a lamellar structure was exhibited instead. 

Regarding microphase separation, Mackay et al. [59] reported that hybrid dendritic block copolymers consisting of poly(benzyl ether) dendron and linear polystyrene, self-assembled on lamellar and cylindrical structures. By taking advantage of a similar system Pochan et al. [60] observed similar morphologies even in low average molecular weights. Both research groups [59,60] indicated that despite the rarely low enthalpic parameter (*χ*), the obtained morphology is an outcome of conformational asymmetry and is strongly affected by the extremely high entropic parameter (degree of polymerization, N). 

Hammond et al. [61] studied the microphase separation in bulk of poly(ethylene oxide) (PEO) final dendritic materials where the PEO chains were appended to a polyamidoamine (PAMAM) dendron. 

Bulk microphase-separation studies of relatively identical amphiphilic dendritic polymers were studied with small-angle X-ray scattering (SAXS) leading to hexagonal and lamellar morphologies [62,63]. 

Through chemically modified block copolymers consisting of dendritic PEO and linear PS chains, hexagonal close packed cylindrical and alternating lamellar morphologies were obtained when studied in bulk [64]. 

In this study, we report the synthesis, and molecular, thermal, and morphological characterization of both symmetric and asymmetric dendritic terpolymers comprising homo-arm star polymers and miktoarm star copolymers as the inner first generation, respectively. The route adopted for the synthesis involves a convergent method, because the outer generation was initially prepared, followed by the inner generation which was prepared by combining chlorosilane chemistry with corresponding active macroinitiator sites. The leading role on the successful synthesis is attributed to the linking agents (4-clorodimethylsililstyrene (CDMSS) [31,32,33,34] and trichloromethylsilane (CH_3_SiCl_3_) or tertachlorosilane (SiCl_4_)).

By employing three chemically different segments, namely PS, PB (exhibiting increased 1,4-microstructure (~90–92%)) and PI (increased 3,4-microstructure (varying from 55 to 60%)), two symmetric samples were synthesized of the following sequences: [(PS)(PI)(PB)]_3_-B_3_ core and [(PS)(PI)(PB)]_4_-B_4_ core, where in each case the PB segment consisted the homo-arm core (with three and/or four arms respectively) and the remaining two blocks constituted the outer generation. 

Moreover, two asymmetric dendritic terpolymers were synthesized and due to the complexity of the structure are denoted as [(PB)(PI)(PS_c_)][(PS)(PB)(PI_c_)]_2_ (SI_2_ core), [(PB)(PI)(PS_c_)][(PS)(PB)(PI_c_)]_3_ (SI_3_ core), where the letter “c” is used in order to demonstrate the block linked to the coupling agent that constitutes the core. 

The initiative for the synthesis of such architectures was to study their morphological behavior due to the increase in the junction points and compare them to the corresponding initial miktoarm terpolymers (dendrons). 

**Figure 1:**
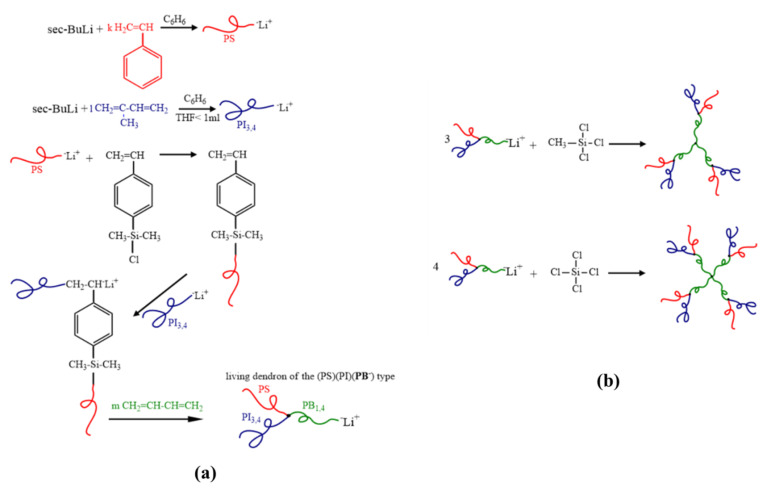
Schematic illustration of: (**a**) the synthetic procedure for the living dendron of the (PS)(PI)(**PB^−^**) type; and (**b**) the coupling reaction for the formation of the final symmetric dendritic terpolymers of the [(PS)(PI)(PB)]_3_ or B_3_ core and [(PS)(PI)(PB)]_4_ or B_4_ core respectively.

**Figure 2:**
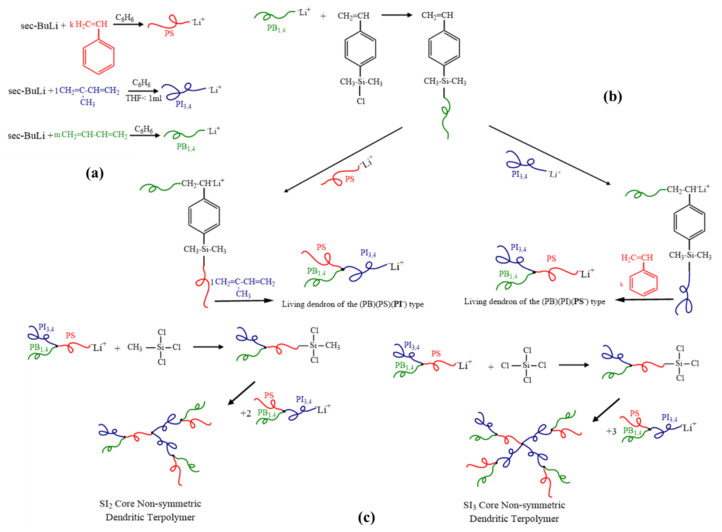
Schematic illustration of: (**a**) the synthetic procedure for the three different PS, PB and PI segments used in the intermediate steps for the synthesis of the two different living dendrons, (**b**) the preparation for the two different living dendrons of the (PB)(PS)(**PI^−^**) and the (PB)(PI)(**PS^−^**) types respectively and (**c**) the coupling reaction for the formation of the [(PB)(PI)(PS_c_)][(PS)(PB)(PI_c_)]_2_ or SI_2_ core type and [(PB)(PI)(PS_c_)][(PS)(PB)(PI_c_)]_3_ or SI_3_ core type non-symmetric dendritic terpolymers.

All reactions leading to precursors, intermediate and final products, concerning [(PS)(PI)(PB)]_3_-B_3_ core and [(PS)(PI)(PB)]_4_-B_4_ core are illustrated in Figure 1 and [(PB)(PI)(PS_c_)][(PS)(PB)(PI_c_)]_2_ (SI_2_ core) and [(PB)(PI)(PS_c_)][(PS)(PB)(PI_c_)]_3_ (SI_3_ core)] are illustrated in Figure 2 and were monitored as well with SEC instrumentation which are presented in the supporting information (Figures S3 and S4). Details concerning the fractionation procedure are given in the Supporting Information.

## 2. Results and Discussion

### 2.1. Synthesis

**Synthesis of symmetric dendritic terpolymers**. The detailed polymer synthesis for the symmetric dendritic terpolymers is already thoroughly mentioned in the literature where the convergent method was used [34]. In all cases, the polymeric chains which correspond to the second generation (outer domains) are initially synthesized and then are linked with the CDMSS in order to create the macroinitiator which will polymerize the third monomer and therefore create the living star terpolymer (dendron). 

The synthesis of [(PS)(PI)(PB)]_3_-B_3_ core and [(PS)(PI)(PB)]_4_-B_4_ core samples was accomplished according to the aforementioned approach and the synthetic details including the amount of used chemicals, yields, fractionation conditions, reaction temperature, reaction time are thoroughly described in our previous work [34]. The living dendrons were combined using methyltrichlorosilane (CH_3_SiCl_3_) or tetrachlorosilane (SiCl_4_) resulting in the final dendritic polymers. Chlorosilane chemistry was exploited in order to achieve the symmetric dendritic polymers. All reactions leading to precursors, intermediate and final products (Figure 1) were monitored via SEC instrumentation and are depicted in the supporting information (Figures S1–S2).

**Synthesis of non-symmetric dendritic terpolymers.** In order to synthesize the non-symmetric dendritic terpolymers, the following procedure was adopted, which is different from the one we used for the symmetric type dendritic terpolymers [34] and is reported for the first time. Initially, a living polymer chain [PB^(−)^Li^(+)^] was reacted selectively with the chlorosilane group of 4-(chlorodimethylsilyl) styrene (0.40 mmol in 10 mL of benzene) and the equivalent macromonomer was prepared. The PB living chain was prepared by using 5 g of butadiene (0.092 mol) and initiator (0.40 mmol *sec*-BuLi) in 150 mL of benzene, which were left to react for 24 h. The synthetic strategy of the macromonomers requires the addition of stoichiometric amounts of the living chain in the solution of the coupling agent. The active macromolecular segments are terminated by the reaction of the living end with the chlorosilyl group and LiCl is produced. Afterwards, the slow addition of the previously synthesized macromonomer into the solution of the second living chain [PS^(−)^Li^(+)^] was conducted, whose functionality leads to the addition of the initial macromonomer through the vinyl bond. The PS living chain was synthesized by mixing 5 g of styrene (0.048 mol) with *sec*-BuLi (0.40 mmol) in 150 mL of benzene and left to react overnight. The substitution of the chlorosilyl group is faster and sterically preferred than the addition towards the vinyl bond, therefore, the second addition is much slower. The necessary amount of the third monomer (isoprene, 5 g or 0.073 mol) is then polymerized leading to the first generation terpolymer dendron. The addition of living chains onto the CDMSS leading to the macromonomer was achieved through an end coupling reaction, where 2–3 monomeric units of styrene were added. Through this approach, the living ends were altered to PS^(−)^Li^(+)^ leading to better control of the linking reaction. In order to improve the kinetics of the coupling reaction, a very small quantity of polar solvent was added (THF < 1 mL) in all cases.

Through the aforementioned synthesis procedure, the (PB)(PS)(PI)^(−)^Li^(+)^ living dendron was prepared. Correspondingly, the synthesis of the living dendron of (PB)(PI)(PS)^(−)^Li^(+)^ type is completed, by altering the sequence between PS and PI chains, leading eventually to the opportunity of combining different type of living dendrons into the final dendritic terpolymer through chlorosilane chemistry by using either CH_3_SiCl_3_ or SiCl_4_.

Significant control of the synthesis and minimal termination reactions leading to undesired products was evident, due to the capping reaction, wherever needed, as it is evident from the molecular characterization results. In Table 1 and Table 2, respectively, the number-average molecular weight (${\bar{M}}_{n}$) of each segment is separately given. Furthermore, in addition to the SEC chromatographs (Figures S1–S4) the ^1^H-NMR spectra are also presented in the supporting information (Figures S5–S8). The molecular characterization results (SEC, MO, ^1^H-NMR) indicated that all initial blocks, intermediate products and final symmetric and non-syvmmetric dendritic terpolymers exhibited narrow distributions (below 1.1) with relatively good yields for the final dendritic structures, ranging from 60% up to 70%, despite their complex architecture. The successful syntheses of all dendritic terpolymers was confirmed through the calculation of the weight fractions per segment from the ^1^H-NMR spectra, together with the relevant microstructures’ estimation of the polydiene chains. It should be noted that, due to the stepwise reactions, which demand numerous protection and linking reactions, the finally produced samples are a mixture of the wanted synthesized asymmetric dendritic terpolymers, along with the unreacted dendrons and all other byproducts, leading to the necessity of extracting all the unnecessary products. This was accomplished by adopting the fractionation technique in order to purify the final dendritic terpolymers. Repeated fractionations were performed until only one peak was evident, corresponding exclusively to the desired final dendritic terpolymers (Figures S1–S4).

### 2.2. Thermal Characterization 

As can be observed from the DSC thermographs, the samples exhibited three and/or four distinctive glass transition temperatures (T_g_), corresponding to all blocks in each sample, suggesting either a three-phase structure during morphological characterization and/or a two-phase system which is attributed to mixed polydienes, respectively. In the case of polydienes (PI and PB), the existence of a methyl group on the monomeric units of the PI blocks leads to an increase in T_g_, and as a result PB_1,4_ (high isomerism 1,4 ~90%) displays a T_g_ ~ −90 °C, while PI_1,4_ (high isomerism 1,4 ~92%) displays a T_g_ ~ −67 °C. The microstructure variations per polydiene block, as well as their contribution in the macromolecular chain, have a great impact on the glass transition temperature, due to differentiations in chain stereochemistry and in flexibility. Specifically, PI_1,4_ (high isomerism 1,4 ~ 100%) has a T_g_ value equal to −70 °C, in contrary to the PI_3,4_ (high isomerism 3,4 ~55–60%), which is more stereochemically hindered and the T_g_ value is equal to −30 °C [65,66]. The same behavior is adopted for the PB block, where the linear PB_1,4_ (high isomerism 1,4 ~90%) has a T_g_ value equal to −90 °C, while branched PB_1,2_ (high isomerism 1,2 ~100%) has a T_g_ value at approximately 0 °C. DSC thermographs for the most of the miktoarm precursors and the final dendritic terpolymers are presented in the supporting information (Figures S9–S12). Specifically, for the samples (B_3_-core and B_4_-core) exhibiting higher percentage of the PB_1,2_ microstructure, an additional distinct T_g_ approximately at −10 °C is observed, possibly indicating the mixing of the polydiene blocks.

### 2.3. TEM and SAXS Results

In order to examine the obtained morphologies of symmetric and non-symmetric dendritic terpolymers, transmission electron microscopy (TEM) and small angle X-ray scattering (SAXS) were employed. The necessary procedures for the preparation of the necessary thin sections for TEM studies have already been reported in the literature [33,65]. Casting in toluene (4% *w*/*v*), which is almost a non-selective solvent for all segments, was performed for all samples (miktoarm precursors and final dendritic terpolymers) [65,67]. Prior to TEM studies, staining with vapors of OsO_4_ diluted in aqueous solution (2% *w*/*v*) was employed for all sections for specific times, in order to differentiate the electron density between the polydienes, as described elsewhere [33,65]. It should be mentioned that the obtained morphologies were examined for unannealed samples, and after thermal annealing at 120 °C for five days. In Table 3, the estimated values for the Flory–Huggins interaction parameters of all possible combinations are presented at 20 °C and 120 °C based on specific calculated equations reported already in the literature [68].

#### 2.3.1. [(PS)(PI)(PB)]_3_-B_3_ core

**Figure 3:**
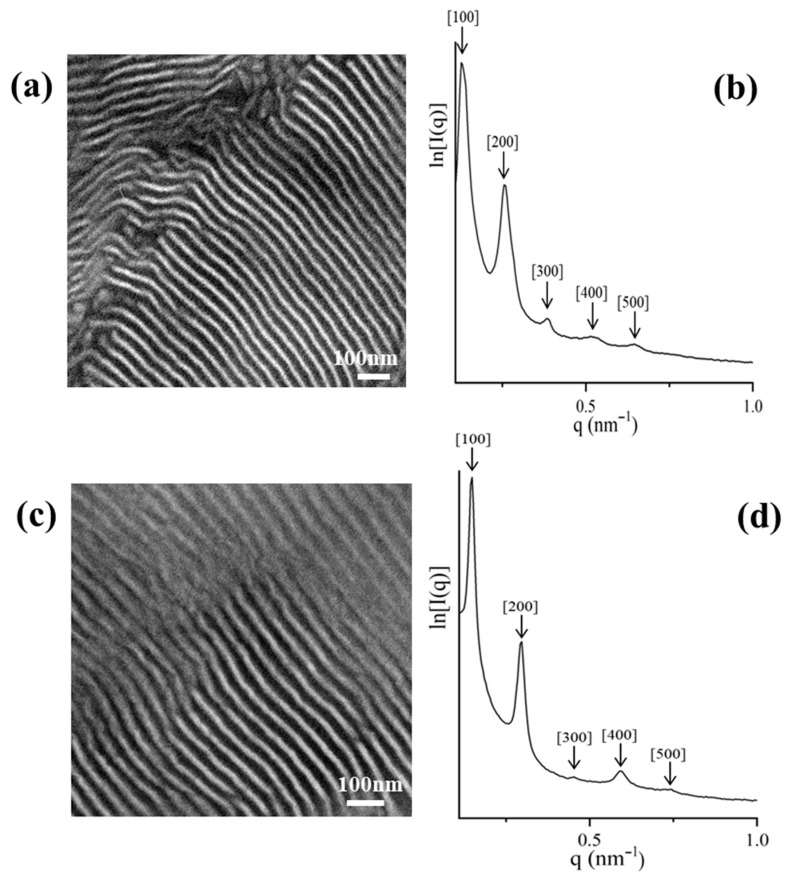
Bright-field transmission electron microscopy (TEM) images of the dendritic final terpolymer [(PS)(PI)(PB)]_3_-B_3_ core and the corresponding dendron where in both cases 2-phase contrast is clearly indicated (**a**,**c**) after staining with vapors of OsO_4_ aqueous solution and their relevant 1D small-angle X-ray scattering (SAXS) plots (**b**,**d**). In both cases, alternating lamellae domains are clearly observed in the TEM images and are verified by the observed peaks ratio at the 1D SAXS plots. In all TEM images, white areas correspond to PS and dark correspond to mixed PI and PB segments.

The morphological characterization results for the symmetric dendritic terpolymer of [(PS)(PI)(PB)]_3_-B_3_ core type, with ${\bar{M}}_{n}^{dendritic}$ = 113 kg/mol and block weight fraction ratio (*w*/*w* %) equal to 37, 31 and 32 for PS, PB and PI, respectively, are presented in comparison to the corresponding miktoarm terpolymer dendron (Figure 3). It should be mentioned that for the completion of the specific sequence, the presence of a polar environment (THF) is necessary in order to obtain the specific PI_3,4_, while for the PB_1,4_, a completely non-polar environment is needed (the two polydienes microphase separate at specific microstructures and percentages as already reported in the literature by Avgeropoulos et al.) [33,65]. Due to the existence of traces of THF during the synthesis of the third segment, an increased percentage of PB_1,2_ microstructure (~26%) is inevitable (as evident from the ^1^H-NMR spectrum, Figure S5 in the supporting information) [69,70,71,72]. Therefore, in this case, the differentiation on the usual 1,4-microstructure percentage for the PB block leads to evident mixing with the PI_3,4_ segments, as can clearly be seen in the TEM images (Figure 3a). Actually, a two-phase morphology, instead of three-phase, exhibiting alternating lamellar domains, is obtained, where the PS bright colored regions occupy approximately one-third of the overall unit cell, while the polydiene domains appear dark and conceive the remaining space of the unit cell. Different grain boundaries are evident in the TEM images and can be assigned to the increased chain flexibility of the polydienes when compared to the PS domains. Further annealing was employed, inducing no severe variations in the aforementioned morphology. 

The relevant SAXS plot (Figure 3b) was in agreement with the TEM results, indicating a substantial number of peaks (five), which conclude in a significantly well-organized film. In the TEM images, it is evident that the adopted lamellae morphology exhibits long range order, which in the SAXS plots will be verified by the observation of an increased number of peaks. Peak positions in the SAXS plot were associated towards the position of the first peak, and the corresponding ratios of *q*_1_/*q*_2_/*q*_3_/*q*_4_/*q*_5_ led to a relevant ratio of approximately 1:2:3:4:5, which is in agreement with the theoretical prediction for alternating lamellar domains (p_n_ space group), and the domain spacing for this dendritic sample is approximately 42 nm.

The morphological results for the initial dendron (star terpolymer precursor of the (PS)(PI)(PB) type) were similar to those of the dendritic terpolymer [(PS)(PI)(PB)]_3_. TEM images (Figure 3c) indicating lamellar morphology of alternating PS (bright color) and miscible PI/PB (dark) layers, exhibited significant long-range order. In accordance with the dendritic terpolymer results, the layer thickness of the mixed polydienes is almost twice the size of the PS, further confirming the sample composition. The values of the observed peaks in the SAXS plot (Figure 3d) correspond to a ratio of 1:2:3:4:5, and the d-spacing is approximately equal to 43 nm. It is straightforward from the TEM and SAXS plots for the dendron and the dendritic terpolymer that the enhancement of junction points (one for the dendron vs. four for the dendritic final terpolymer) and the increase in the number-average molecular weight by a factor of three (in the dendritic sample) did not affect the domain spacing dimensions.

**Figure 4:**
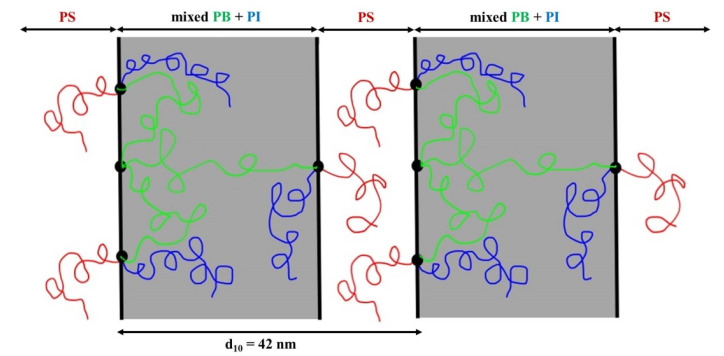
Schematic illustration of the different segments’ arrangement of the [(PS)(PI)(PB)]_3_-B_3_ core type dendritic terpolymer.

The complex architecture did not substantially change the d-spacing, despite the adopted different conformational entropy. A schematic of the segments’ arrangement for the specific dendritic sample is illustrated in Figure 4, where the PB core is actually either looping at the interface and the two chains evidently conform an entropic penalty, whereas the one PB chain which may be bridged between two different interfaces also leads to entropic effects due to topology constraints. The result of this behavior is the lateral expansion of the intermediate dividing surface (IMDS), where all the junction points are located, and the adaptation of significant order in the complex architecture is evident (from the TEM images and the five peaks from the 1D SAXS plot) but unfortunately the two polydienes are miscible due to the differentiation of the geometric isomerisms compared to those reported already in the literature [51,67].

#### 2.3.2. [(PS)(PI)(PB)]_4_-B_4_ core

**Figure 5:**
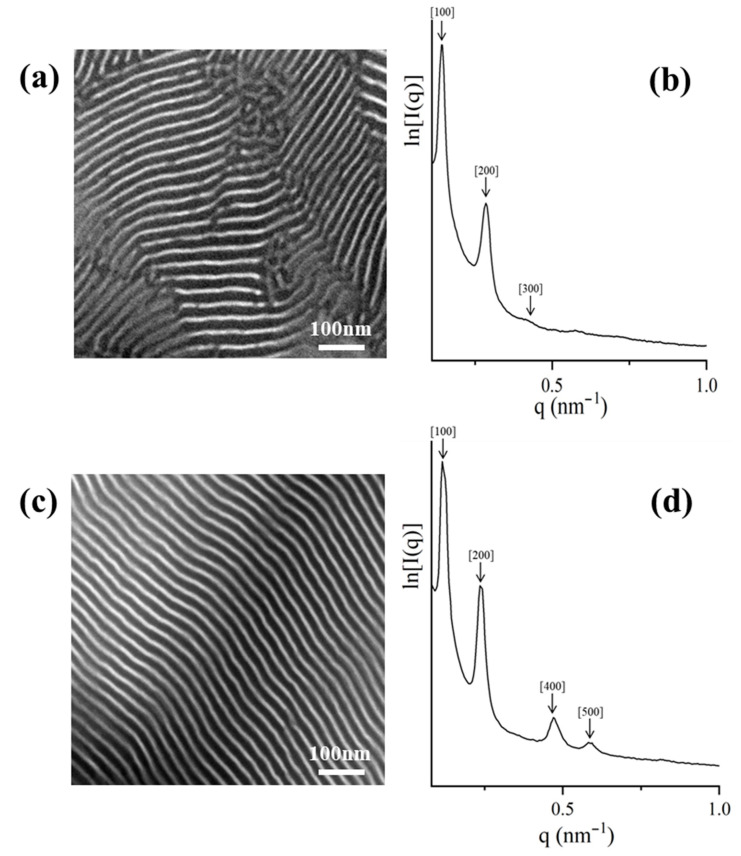
Bright-field TEM images of the dendritic final terpolymer [(PS)(PI)(PB)]_4_-B_4_ core and the corresponding dendron where in both cases 2-phase contrast is clearly indicated (**a**,**c**) after staining with vapors of OsO_4_ aqueous solution and their relevant 1D SAXS plots (**b**,**d**). In both cases, alternating lamellae domains are clearly observed in the TEM images and are verified by the observed peaks ratio at the 1D SAXS plots. In all TEM images white areas correspond to PS and dark to mixed PI and PB segments.

[(PS)(PI)(PB)]_4_-B_4_ core type, with ${\bar{M}}_{n}^{dendritic}$ = 192 kg/mol and block volume fraction ratio (%) equal to 28, 34 and 38 for PB, PS and PI, respectively, and their corresponding dendrons (different from that used for the B_3_ core sample above) were characterized through TEM and SAXS. Similar to what has been mentioned for the previous sample, due to the synthetic procedure, an increased percentage of PB_1,2_ microstructure (~20%) is evident at the ^1^H-NMR spectrum (Figure S6 in the supporting information). A two-phase system is observed from the TEM images (Figure 5a), where alternating lamellae are again evident with PS white domains and mixed polydienes dark segments. Due to the increased stereochemical hindrance, induced by the introduction of an extra dendron to the system, a larger number of macromolecular chains must be arranged, therefore increasing the grain boundaries as evident from the TEM studies. Thermal annealing at 120 °C for five days did not alter the obtained morphology, indicating that the adopted lamellar structure constitutes equilibrium morphology. The increased number of grain boundaries led to only two permitted peaks from the SAXS plot (Figure 5b), corresponding to a ratio of 1:2:-:4:5, further confirming the lamellar morphology with a calculated d-spacing equal to 54 nm. The corresponding (PS)(PI)(PB) miktoarm terpolymer (dendron) showed a similar behavior during the morphological studies with the dendritic terpolymer consisting only one-quarter of the overall dendritic composition. In the dendron, the PB also exhibited an increased percentage of the PB_1,2_ microstructure (~20%), causing again the miscibility among the two polydienes forming a two-phase system as in the case of the complex dendritic terpolymer (Figure 5c). The permitted peaks extracted from the SAXS plot (Figure 5d), correspond to a ratio of 1:2:3, further confirming the lamellar morphology with d-spacing estimated equal to 45 nm. As already mentioned above, for the B_3_ core dendritic sample and its corresponding dendron, in the case of the B_4_ final complex sample from the TEM and SAXS plots, the enhancement of junction points (one for the dendron vs. five for the dendritic final terpolymer) and the increase in the number-average molecular weight by a factor of four (in the dendritic sample) did not affect the domain spacing dimensions. A similar explanation to that mentioned above based on entropy constrains can be also adopted.

#### 2.3.3. [(PB)(PI)(PS_c_)][(PS)(PB)(PI_c_)]_2_ (SI_2_ core)

**Figure 6:**
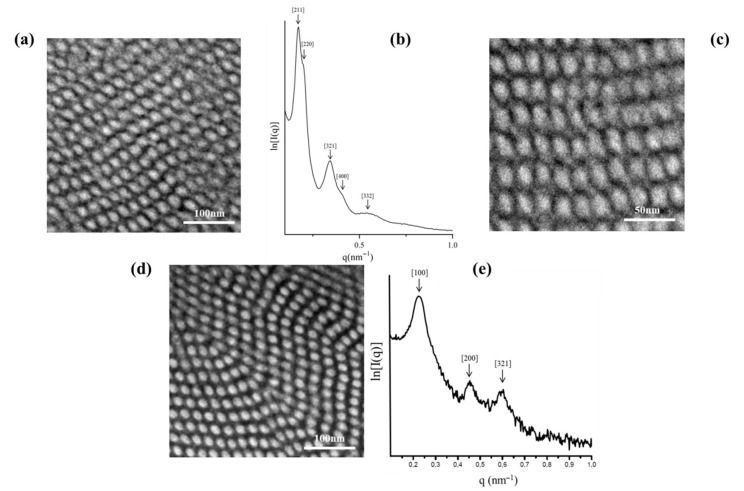
Bright-field TEM images of the dendritic final terpolymer [(PB)(PI)(PS_c_)][(PS)(PB)(PI_c_)]_2_ (SI_2_ core), where 3-phase contrast is clearly indicated (**a**,**c**) after staining with vapors of OsO_4_ aqueous solution and the relevant 1D SAXS plots (**b**). Cubic domains are observed in the TEM images (**a**,**c**) exhibiting almost [111] and [100] high symmetry projections which are verified by the observed peaks ratio at the 1D SAXS plots. In all TEM images, white areas correspond to PS, grey to PI, and dark to PB segments. Bright-field TEM image of the corresponding dendron (consisting only one-third of the final dendritic terpolymer and the relevant 1D SAXS plots are shown (**d**,**e**) where a square packing of black (PB) and white (PS) cylinders in a grey matrix (PI) are observed in the TEM and the $\sqrt{1}:\sqrt{4}:\sqrt{7}$ ratio from the SAXS plot justifies the cylindrical domains.

Bulk morphological characterization of [(PB)(PI)(PS_c_)][(PS)(PB)(PI_c_)]_2_ (SI_2_ core) dendritic terpolymer exhibiting ${\bar{M}}_{n}^{\mathrm{dendritic}}$ = 151 kg/mol and block volume fraction ratio (%) equal to 35, 36 and 29 for PB, PS and PI, respectively, indicated a cubic structure (Figure 6a,c), where the PS blocks (white areas) and PB segments (black areas) formed two continuous, three-dimensional, periodic, interpenetrated, but not interconnected, networks inside a PI matrix (grey areas). This is the very first time, to the best of our knowledge, that an ordered cubic structure has been observed in such complex asymmetric dendritic terpolymers exhibiting a high number-average molecular weight. 

The 1D SAXS plot (Figure 6b) for the specific sample leads to the conclusion that the cubic structure is more consistent with the double gyroid (DG) topology, because the first two reflections correspond to the $\sqrt{3}\;:\sqrt{4}\;$ratio, as already reported in the literature for simpler architectures by the Avgeropoulos group [65,73]. In Figure 6b, a significant number of permitted reflections are missing and may be attributed to the complexity of the final dendritic material in combination with the increased total number-average molecular weight. Actually, based on the literature [65,73] the ratio of the permitted reflections for the DG structure is: $\sqrt{3}:\sqrt{4}:\sqrt{7}:\sqrt{8}:\sqrt{11}:\sqrt{12}:\sqrt{13}\ldots .$. From the SAXS plot for the dendritic terpolymer, the d-spacing was calculated at approximately equal to 36 nm. It is the first time that a second generation dendritic terpolymer has been studied in which the first generation is a 3-miktoarm star copolymer of the PS(PI)_2_ sequence. A major conclusion that can be extracted from studying this material is that complexity in architecture signifies the entropic and enthalpic constrains in order for an equilibrium structure to be derived. 

TEM experiments (Figure 6d) on the one dendron of the (PB)(PI)(**PS^−^**) miktoarm star terpolymer, which is used as a precursor for the asymmetric dendrimer, consisting of one-third of the overall composition, indicated a cylindrical morphology after staining with OsO_4_ vapors. Specifically, four PS cylinders (white), surrounding a PB cylinder (black) inside a PI matrix (grey color) are observed, indicating a square packing of the PS/PB cylinders, which is a morphology expected and already observed in linear triblock terpolymers as mentioned in the literature [74,75]. The relative *q* values of the observed peaks q_1_:q_2_:q_3_ from the SAXS plot (Figure 6e) correspond to a ratio of $\sqrt{1}:\sqrt{4}:\sqrt{7}$, and the d-spacing is equal to 28 nm, a value not significantly smaller from that calculated for the final complex material. 

The miktoarm terpolymer, which comprised two-thirds of the overall morphology, was not feasibly isolated, therefore, no TEM and SAXS results could be obtained for this specific dendron. 

#### 2.3.4. [(PB)(PI)(PS_c_)][(PS)(PB)(PI_c_)]_3_ (SI_3_ core)

**Figure 7:**
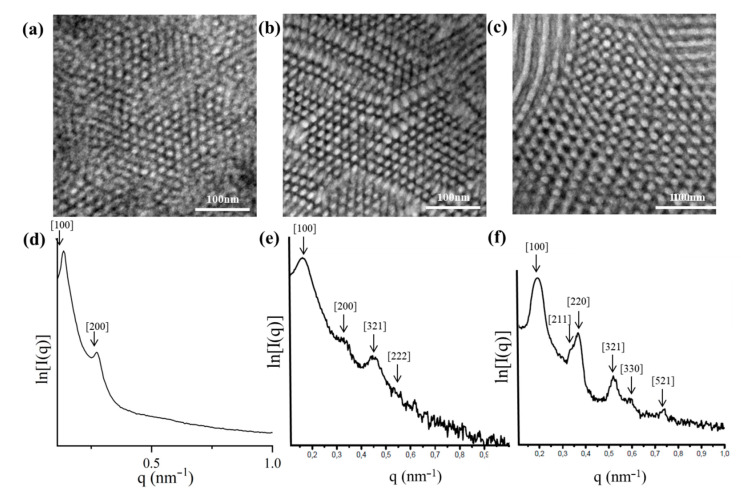
Bright-field TEM images of the dendritic final terpolymer [(PB)(PI)(PS_c_)][(PS)(PB)(PI_c_)]_3_ (SI_3_ core) and the corresponding dendrons (consisting one-quarter and three-quarters of the final dendritic terpolymer), where 3-phase contrast is clearly indicated (**a**,**b**,**c**) after staining with vapors of OsO_4_ aqueous solution and their relevant 1D SAXS plots (**d**,**e**,**f**). PS (white) microdomains are ordered on a tertiary symmetry axis, surrounded by four PB (black) microdomains in a (grey) PI matrix. This morphology resembles the cross-section of cylindrical morphologies and the order is best on the dendrons and worst in the dendritic terpolymer, which can be verified by the number of peaks for the corresponding SAXS plots.

The non-symmetric dendritic terpolymer with four dendrons (three identical and one different) of the [(PB)(PI)(PS_c_)][(PS)(PB)(PI_c_)]_3_ (SI_3_ core) type, with ${\bar{M}}_{n}^{\mathrm{dendritic}}$ = 214 kg/mol and block volume fraction ratio (%) equal to 25, 35 and 40 for PB, PS and PI, respectively, did not conclude in a well-ordered structure as illustrated in the TEM images (Figure 7a) when compared to the sample [(PB)(PI)(PS_c_)][(PS)(PB)(PI_c_)]_2_ (SI_2_ core) described above. PS (white) microdomains are ordered on a tertiary symmetry axis, surrounded by four PB (black) microdomains in a (grey) PI matrix. This morphology resembles the cross-section of cylindrical morphologies predicted for star terpolymers [76,77]. The SAXS plot (Figure 7b) indicates a not well-ordered structure; only two reflections are evident, leading to a ratio of 1:2. From the first reflection, it was possible to calculate the d-spacing of the dendritic material as equal to 46 nm. 

In order to synthesize the specific non-symmetric dendritic terpolymer two different miktoarm terpolymers were used: (PB)(PI)(**PS^−^**) that constituted one-quarter of the overall architecture and (PS)(PB)(**PI^−^**) which constituted three-quarters of the overall structure. The miktoarm terpolymer of the (PB)(PI)(**PS^−^**) type when studied with TEM (Figure 7c), presented a PI matrix where well-ordered PS and PB cylinders in square lattices are evident, while the (PS)(PB)(**PI^−^**) showed a similar morphology but not as well-ordered, as illustrated in Figure 7b. 

The values of the permitted reflections from the SAXS plot (Figure 7e) for the (PB)(PI)(**PS^−^**) precursor correspond to a ratio of $\sqrt{1}:\sqrt{4}:\sqrt{7}:\sqrt{12}$, and the d-spacing is approximately equal to 39 nm. The absence of $\sqrt{3}:\sqrt{9}$ can be probably attributed to the structure factor. For the (PS)(PB)(**PI^−^**) precursor, the values of the observed peaks correspond to a ratio of $\sqrt{1}:\sqrt{3}:\sqrt{4}:\sqrt{7}:\sqrt{9}:\sqrt{15}$, and the d-spacing is equivalent to 33 nm. In both dendron cases, the hexagonally close packed (hcp) cylindrical morphologies are verified by the SAXS plots, and well-ordered structures may be concluded based on the large number of permitted reflections on the specific 1D plots.

## 3. Materials and Methods

**Materials**. Analytical information on the high vacuum technique, as well as the purification procedures for the monomers (butadiene, isoprene, styrene), solvents (benzene, toluene, tetrahydrofuran (THF)), initiator (*sec*-BuLi), coupling agents (trichloromethylsilane, tetrachlorosilane) to the standards required for anionic polymerization are well known and are mentioned elsewhere [31,32,33,34]. The 4-(Chlrodimethylsilyl) styrene was synthesized from p-chlorostyrene and dichlorodimethylsilane through a Grignard reaction, under high vacuum techniques as already described elsewhere in detail [31,32,33,34]. 

**Instrumentation**. The number-average molecular weights (${\bar{M}}_{n}$) (higher than 15,000 g/mol) of the precursors and the final products were measured with a membrane osmometer (MO) Gonotec-Osmomat 090 at 35 °C. Number-average molecular weights (${\bar{M}}_{n}$) (lower than 15,000 g/mol) of the precursors were measured with a vapor pressure osmometer (VPO) Gonotec-Osmomat 070 at 45 °C, which was calibrated with a benzyl solution to determine the consistency and accuracy of the instrument. For both measurements, toluene was used after distillation over CaH_2_ as a solvent. 

Size exclusion chromatography (SEC) employing a PL-GPC 50 Integrated GPC System from Agilent Technologies, was calibrated with eight PS standards M_p_: 4.3 kg/mol to 3000 kg/mol, and prior to calculating the polydispersity indices of the unknown materials a series of standard PS solutions were tested in order to also examine the accuracy of the instrumentation.

Proton nuclear magnetic resonance spectroscopy (^1^H-NMR) was used for the determination of the block composition and the polydiene microstructure of the final materials and their precursors. The experiments were carried out in CDCl_3_ at 30 °C using a Bruker AVANCE II spectrometer and the data were processed using UXNMR (Bruker) software (Bruker GmbH, Berlin, Germany).

Differential scanning calorimetry (DSC) experiments, employing a TA Instruments Q100 Modulated DSC, (TA Instruments Ltd., Leatherhead, England) were used in order to obtain preliminary information concerning the behavior of the three components by evaluating specific thermal transitions, because three different glass transition temperatures were exhibited, being a first indication for the three-phase microphase separation. The values were: T_g1_~−90 °C (for the PB chains), T_g2_~−27 °C (for the PI enriched in 3,4-microstrucure), and T_g3_~100 °C (for the PS chains).

Transmission electron microscopy (TEM) and small-angle X-ray scattering (SAXS) were employed for the morphological characterization of the dendrons and the final dendritic terpolymers. More details on the setup of these specific instruments are given elsewhere [65]. For the dendrons used as precursors for the asymmetric final materials, a different SAXS setup was employed, more specifically, a Molecular Metrology ASSY 610-004378, U.S.A. system. It is important to mention that because the molecular characteristics are relatively high, and in general the dendrons and the final dendritic terpolymers when self-assembled in bulk are well above the conditions of the weak segregation regime, the dried films, after solvent evaporation through casting, were annealed for 5 days at 120 °C under vacuum in order to obtain near-equilibrium microstructures. For TEM investigation, 500–1000 Å thick sections were cryomicrotomed at −90 °C and the sections were picked up on 600-mesh copper grids. The grids were then placed in the vapors of a 4% OsO_4_–water solution for selective staining of the two polydiene domains.

## 4. Conclusions 

In this work, anionic polymerization was employed in order to control the design of the final well-defined complex macromolecules, exhibiting low polydispersities, leading to core-symmetric or non-symmetric materials with potentially improved applications. The symmetric dendritic terpolymers were synthesized from three or four miktoarm star terpolymers, exhibiting identical chemical composition, which were linked together from the same block through chlorosilane chemistry reactions. Accordingly, asymmetric dendritic terpolymers were synthesized, deriving from two different miktoarm star terpolymers, linked onto the central linking agent through a chemically different chain in order to create the SI_2_ and SI_3_ core sequences. The successful synthesis was verified through SEC, MO, VPO, and DSC, and morphological characterization was achieved via TEM and SAXS. The morphological characterization results for the symmetric dendritic terpolymers led to the conclusion that, due to the mixing between the specific PI_3,4_ and the PB blocks (attributed to the increased percentage of 1,2-microstructure for the PB segments) two phase systems were observed from the TEM studies. Self-assembled three phase structures were obtained despite the architecture complexity of the non-symmetric dendritic terpolymers. Due to the thermodynamically favorable arrangement of four and five junction points for the SI_2_ and SI_3_ core dendritic polymers, respectively, the curvature of the interfaces upon which these junction points are arranged, may lead to increased number of defects and/or dislocations of the adopted final morphologies.

## Figures and Tables

**Table 1:** Molecular characteristics of the symmetric dendritic terpolymers calculated by membrane osmometry/ vapor pressure osmometry (MO/VPO) and size exclusion chromatography SEC.

Symmetric Terpolymers						
Dendritic Sample	${\bar{M}}_{n}^{PS}$ kg/mol ^a^	${\bar{M}}_{n}^{PB}$ kg/mol ^a^	${\bar{M}}_{n}^{PI}$ kg/mol ^a,b^	${\bar{M}}_{n}^{dendron}$ kg/mol ^b^	${\bar{M}}_{n}^{dendritic}$ kg/mol ^b^	Đ ^c^
B_3_-core	13.0	13.4	11.3	37.7	113.1	1.05
B_4_-core	14.4	18.0	15.7	48.1	192.4	1.08

^a^ Calculated from the results from VPO in toluene at 45 °C and SEC. ^b^ MO membrane osmometry in toluene at 35 °C. ^c^ SEC in THF at 30 °C with PS standards.

**Table 2:** Molecular Characteristics of the asymmetric dendritic terpolymers calculated by MO/VPO and SEC.

Asymmetric Terpolymers							
Dendritic Sample	“Living Dendron”	${\bar{M}}_{n}^{PS}$ kg/mol ^a^	${\bar{M}}_{n}^{PB}$ kg/mol ^a^	${\bar{M}}_{n}^{PI}$ kg/mol ^a^	${\bar{M}}_{n}^{star}$ kg/mol ^a^	${\bar{M}}_{n}^{tot}$ kg/mol ^b^	Đ ^c^
(SI_2_)-core	1 × (PB)(PI)(**PS^−^**)	50.0	11.6	15.6	77.2	141.2	1.07
	2 × (PS)(PB)(**PI^−^**)	12.0	10.0	10.0	32.0		
(SI_3_)-core	1 × (PB)(PI)(**PS^−^**)	40.0	13.3	14.5	67.8	214.0	1.06
	3 × (PS)(PB)(**PI^−^**)	12.5	12.2	25.0	48.7		

^a^ Calculated from the results from VPO in toluene at 45 °C and SEC. ^b^ MO membrane osmometry in toluene at 35 °C. ^c^ SEC in THF at 30 °C with PS standards.

**Table 3:** Flory–Huggins interaction parameters for all block combinations.

*χ*	PS	1,4-PB	1,4-PI	3,4-PI
**1,4-PB**	0.0483 ^a^	0		
**1,4-PI**	0.0706 ^a^	0.0012 ^b^	0	
**3,4-PI**	0.1109 ^a^	0.0105 ^b^	0.0052 ^b^	0

^a^ 120 °C and ^b^ 20 °C.

## Supplementary Materials

The following are available online. Figure S1: SEC chromatographs of: (A) the initial homopolymer PS, (B) the initial homopolymer PI, (C) synthesized CDMSS macro-initiator during linking reaction process, (D) final unfractionated dendritic terpolymer, (E) fractionated miktoarm dendron of (PS)(PI)(PB^-^) type and (F) fractionated symmetric dendritic terpolymer of [(PS)(PI)(PB)]_3_-B_3_ core type, Figure S2: SEC chromatographs of: (A) the initial homopolymer PS, (B) the initial homopolymer PI, (C) synthesized CDMSS macro-initiator during linking reaction process, (D) unfractionated symmetric dendritic terpolymer, (E) fractionated miktoarm dendron of (PS)(PI)(PB^−^) type and (F) fractionated symmetric dendritic terpolymer of [(PS)(PI)(PB)]_4_-B_4_ core type, Figure S3: SEC chromatographs of: (A) miktoarm dendron of the (PB)(PS)(PI^−^) type, (B) miktoarm dendron of the (PB)(PI)(PS^−^) type, (C) living miktoarm dendron of (PB)(PI)(PS^-^) type after coupling reaction with the CH_3_SiCl_3_, (D) unfractionated asymmetric dendritic terpolymer, (E) fractionated asymmetric dendritic terpolymer of [(PB)(PI)(PSc)]-[(PS)(PB)(PIc)]_2_ type, Figure S4: SEC chromatographs of: (A) miktoarm dendron of the (PB)(PS)(PI^−^) type, (B) miktoarm dendron of the (PB)(PI)(PS^−^) type, (C) living miktoarm dendron of (PB)(PS)(PI^−^) type after coupling reaction with the SiCl_4_, (D) unfractionated asymmetric dendritic terpolymer, (E) fractionated asymmetric dendritic terpolymer of [(PB)(PI)(PSc)]-[(PS)(PB)(PIc)]_3_ type, Table S1: The type and number of protons per monomeric unit of each block as well as the chemical shifts, are presented in order to comprehend the ^1^H-NMR spectra of the dendritic terpolymers, Table S2: Block volume fraction ratios, as well as the geometric isomerism percentage of each polydiene are presented for all dendrons and final dendritic terpolymers as calculated through ^1^H-NMR spectra, Figure S5: ^1^H-NMR spectra of a.) the miktoarm dendron precursor of the [(PS)(PI)(PB^−^)] type and b.) the symmetric dendritic terpolymer of the (PS)(PI)(PB)]_3_-B_3_ core type, Figure S6: ^1^H-NMR spectra of a.) the miktoarm dendron precursor of the [(PS)(PI)(PB^-^)] type and b.) the symmetric dendritic terpolymer of the (PS)(PI)(PB)]_4_-B_4_ core type, Figure S7: ^1^H-NMR spectra of a.) the miktoarm dendron precursor of the (PB)(PI)(PS^−^) type and b.) the asymmetric dendritic terpolymer of the [(PB)(PI)(PSc)]-[(PS)(PB)(PIc)]_2_ type, Figure S8: ^1^H-NMR spectra of a.) the miktoarm dendron precursor of the (PB)(PI)(PS^-^) type, b.) the miktoarm dendron precursor of the (PS)(PB)(PI^−^) type and c.) the asymmetric dendritic terpolymer of [(PB)(PI)(PSc)]-[(PS)(PB)(PIc)]_3_ type, Figure S9: DSC thermograph of the (PS)(PI)(PB^-^) miktoarm dendron precursor (dash line) and the symmetric [(PS)(PI)(PB)]_3_-B_3_ core dendritic terpolymer (solid line), Figure S10: DSC thermograph of the (PS)(PI)(PB^−^) miktoarm dendron precursor (dash line) and the symmetric [(PS)(PI)(PB)]_4_-B_4_ core dendritic terpolymer (solid line), Figure S11: DSC thermograph of the (PB)(PI)(PS^-^) miktoarm dendron precursor (dash line) and the asymmetric [(PB)(PI)(PSc)]-[(PS)(PB)(PIc)]_2_ dendritic terpolymer (solid line), Figure S12: DSC thermograph of the (PS)(PB)(PI^−^) miktoarm dendron precursor (dash line), (PB)(PI)(PS^-^) miktoarm dendron precursor (dash-dot line) and the asymmetric [(PB)(PI)(PSc)]-[(PS)(PB)(PIc)]_3_ dendritic terpolymer (solid line), Table S3: Glass transition temperatures (T_g_) of all synthesized materials.

## References

[1] Schaefgen J.R., Flory P.J. (1948). Synthesis of Multichain Polymers and Investigation of their Viscosities. J. Am. Chem. Soc..

[2] Morton M., Helminiak T.E., Gadkary S.D., Bueche F. (1962). Preparation and Properties of Monodisperse Branched Polystyrene. J. Polym. Sci..

[3] Hadjichristidis N., Roovers J. (1974). Synthesis and Solution Properties of Linear, Four-Branched, and Six-Branched Star Polyisoprenes. J. Polym. Sci. Polym. Phys. Ed..

[4] Hadjichristidis N., Guyot A., Fetters L.J. (1978). Star-branched Polymers. 1. The Synthesis of Star Polyisoprenes Using Octa-and Dodecachlorosilanes as Linking Agents. Macromolecules.

[5] Bauer B.J., Hadjichristidis N., Fetters L.J., Roovers J. (1980). Starbranched Polymers. 5. The Theta Temperature Depression for 8-and 12-arm Polyisoprenes in Dioxane. J. Am. Chem. Soc..

[6] Roovers J., Hadjichristidis N., Fetters L.J. (1983). Analysis and Dilute Solution Properties of 12-and 18-arm-star Polystyrenes. Macromolecules.

[7] Roovers J., Zhou L.L., Toporowski P.M., van der Zwan M., Iatrou H., Hadjichristidis N. (1993). Regular Star Polymers with 64 and 128 Arms. Models for Polymeric Micelles. Macromolecules.

[8] Liu N., Ma C.-H., Sun R.-W., Huang J., Li C.-L., Wu Z.-Q. (2016). Facile synthesis of well-defined ABC miktoarm star terpolymers bearing poly(ε-caprolactone), polystyrene and stereoregular helical poly(phenyl isocyanide) blocks. Polym. Chem..

[9] Chernyy S., Kirkensgaard J.J.K., Mahalik J.P., Kim H., Arras M.M.L., Kumar R., Sumpter B.G., Smith G.S., Mortensen K., Russell T.P. (2018). Bulk and Surface Morphologies of ABC Miktoarm Star Terpolymers Composed of PDMS, PI, and PMMA Arms. Macromolecules.

[10] Li Y., von der Lühe M., Schacher F.H., Ling J. (2018). 3-Miktoarm Star Terpolymers via Janus Polymerization: One-Step Synthesis and Self-Assembly. Macromolecules.

[11] Kim H., Arras M.M.L., Mahalik J.P., Wang W.Y., Yu D.M., Chernyy S., Goswami M., Kumar R., Sumpter B.G., Hong K.L. (2018). Studies on the 3-lamellar morphology of miktoarm terpolymers. Macromolecules.

[12] Yang Y.L., Tsao H.K., Sheng Y.J. (2020). Morphology and Wetting Stability of Nanofilms of ABC Miktoarm Star Terpolymers. Macromolecules.

[13] Ntetsikas K., Zapsas G., Bilalis P., Gnanou Y., Feng X., Edwin L., Thomas E.L., Hadjichristidis N. (2020). Complex Star Architectures of Well-Defined Polyethylene-Based Co/Terpolymers. Macromolecules.

[14] Liu G., Sun H., Rangou S., Ntetsikas K., Avgeropoulos A., Wang S.-Q. (2013). Studying the Origin of “Strain Hardening”: Basic Difference between Extension and Shear. J. Rheol..

[15] Sun H., Ntetsikas K., Avgeropoulos A., Wang S.-Q. (2013). Breakdown of Time-Temperature Equivalence in Startup Uniaxial Extension of Entangled Polymer Melts. Macromolecules.

[16] Sun H., Liu G., Ntetsikas K., Avgeropoulos A., Wang S.-Q. (2014). Rheology of Entangled Polymers Not Far above Glass Transition Temperature: Transient Elasticity and Intersegmental Viscous Stress. Macromolecules.

[17] Sun H., Lin P., Liu G., Ntetsikas K., Misichronis K., Kang N., Liu J., Avgeropoulos A., Mays J., Wang S.-Q. (2015). Failure Behavior After Stepwise Uniaxial Extension of Entangled Polymer Melts. J. Rheol..

[18] Rahman M.S., Aggarwal R., Larson R.G., Dealy J.M., Mays J.W. (2008). Synthesis and Dilute Solution Properties of Well-Defined H Shaped Polybutadienes. Macromolecules.

[19] Chen X., Rahman M.S., Lee H., Mays J.W., Chang T., Larson R. (2011). Combined Synthesis, TGIC Characterization, and Rheological Measurement and Prediction of Symmetric H Polybutadienes and Their Blends with Linear and Star-Shaped Polybutadienes. Macromolecules.

[20] Rahman M.S., Lee H., Chen X., Chang T., Larson R., Mays J.W. (2012). Model Branched Polymers: Synthesis and Characterization of Asymmetric H-Shaped Polybutadienes. ACS Macro Lett..

[21] Chen X., Lee H., Rahman M.S., Chang T., Mays J.W., Larson R. (2012). Analytical Rheology of Asymmetric H-Shaped Model Polybutadiene Melts. Macromolecules.

[22] Roovers J., Toporowski P.M. (1981). Preparation and Characterization of H-Shaped Polystyrene. Macromolecules.

[23] Iatrou H., Hadjichristidis N. (1992). Synthesis of a Model S-Miktoarm Star Terpolymer. Macromolecules.

[24] Tomalia D.A., Dewald J.R., Hall M.R., Martin S.J., Smith P.B. (1984). Reprints of the 1st SPSJ International Polymer Conference, Kyoto, Japan. Soc. Polym. Sci..

[25] Tomalia D.A., Baker H., Dewald J., Hall M., Kallos G., Martin S., Roeck J., Ryder J., Smith P. (1985). A New Class of Polymers: Starburst-Dendritic. Macromol. Polym. J..

[26] Hawker C., Fréchet J.M.J. (1990). Preparation of Polymers with Controlled Molecular Architecture. A New Convergent Approach to Dendritic Macromolecules. J. Am. Chem. Soc..

[27] Bosman A.W., Janssen H.M., Meijer E.W. (1999). About Dendrimers: Structure, Physical Properties, and Applications. Chem. Rev..

[28] Six J.L., Gnanou Y. (1995). From Star-Shaped to Dendritic Poly(ethylene oxide)s: Toward Increasingly Branched Architectures by Anionic Polymerization. Macromol. Symp..

[29] Feng X., Taton D., Chaikof E.L., Gnanou Y. (2005). Toward an Easy Access to Dendrimer-Like Poly(ethylene oxide)s. J. Am. Chem. Soc..

[30] Trollsås M., Hedrick J.L. (1998). Dendrimer-Like Star Polymers. J. Am. Chem. Soc..

[31] Chalari I., Hadjichristidis N. (2002). Synthesis of well-defined second-generation dendritic polymers of isoprene (I) and styrene (S): (S_2_I)_3_, (SI′I)_3_, (I″I′I)_3_, and (I′_2_I)_4_. J. Polym. Sci. A Polym. Chem..

[32] Rangou S., Theodorakis P.E., Gergidis L.N., Avgeropoulos A., Efthymiopoulos P., Smyrnaios D., Kosmas M., Vlahos C. (2007). Synthesis, molecular characterization and theoretical study of first generation dendritic homopolymers of butadiene and isoprene with different microstructures. Polymer.

[33] Avgeropoulos A., Rangou S., Krikorian V., Thomas E.L. (2008). Synthesis and Self-Assembly of 2nd Generation Dendritic Homopolymers and Copolymers of Polydienes with Different Isomeric Microstructures. Macromol. Symp..

[34] Rangou S., Avgeropoulos A. (2009). Synthesis of Dendritic Terpolymers Consisting of Polystyrene, Polybutadiene, and Polyisoprene with Different Isomerisms. J. Polym. Sci. A Polym. Chem..

[35] Urbani C.N., Bell C.A., Lonsdale D., Whittaker M.R., Monteiro M.J. (2008). Self-Assembly of Amphiphilic Polymeric Dendrimers Synthesized with Selective Degradable Linkages. Macromolecules.

[36] Lepoittevin B., Matmour R., Francis R., Taton D., Gnanou Y. (2005). Synthesis of Dendrimer-Like Polystyrene by Atom Transfer Radical Polymerization and Investigation of their Viscosity Behavior. Macromolecules.

[37] Frechet J.M.J., Tomalia D.A. (2002). Dendrimers and Other Dendritic Polymers.

[38] Roovers J., Comanita B. (1999). Dendrimers and Dendrimer-Polymer Hybrids. Advances in Polymer Science.

[39] Tomalia D.A., Frechet J.M.J. (2002). Discovery of dendrimers and dendritic polymers: A brief historical perspective. J. Polym. Sci. A Polym. Chem..

[40] Jang W.-D., Selim K.M.K., Lee C.-H., Kang I.-K. (2009). Bioinsired application of dendrimers: From bio-minicry to biomedical applications. Prog. Pol. Sci..

[41] Svenson S., Tomalia A.D. (2005). Dendrimers in biomedical applications-reflections on the field. Adv. Drug Deliv. Rev..

[42] Lotocki V., Kakkar A. (2020). Miktoarm Star Polymers: Branched Architectures in Drug Delivery. Pharmaceutics.

[43] Astruc D., Charda F. (2001). Dendritic Catalysts and Dendrimers in Catalysis. Chem. Rev..

[44] Yamamoto K., Imaoka T., Tanabe M., Kambe T. (2020). New Horizon of Nanoparticle and Cluster Catalysis with Dendrimers. Chem. Rev..

[45] Kovvali A.S., Chen H., Sirkar K.K. (2000). Dendrimer Membranes: A CO_2_-Selective Molecular Gate. J. Am. Chem. Soc..

[46] Parrott M.C., Benhabbour S.R., Saab C., Lemon J.A., Parker S., Valliant J.F., Adronov A. (2009). Synthesis, Radiolabeling, and Bio-imaging of High Generation Polyester Dendrimers. J. Am. Chem. Soc..

[47] Caminade A.-M. (2020). Phosphorus Dendrimers as Nanotools against Cancers. molecules.

[48] Iatrou H., Avgeropoulos A., Hadjichristidis N. (1994). Synthesis of Model Super H-Shaped Block Copolymers. Macromolecules.

[49] Avgeropoulos A., Poulos Y., Hadjichristidis N., Roovers J. (1996). Synthesis of Model 16-Miktoarm (Vergina) Star Copolymers of the A_8_B_8_ Type. Macromolecules.

[50] Avgeropoulos A., Dair B.J., Hadjichristidis N., Thomas E.L. (1997). Tricontinuous Double Gyroid Cubic Phase in Triblock Copolymers of the ABA Type. Macromolecules.

[51] Avgeropoulos A., Paraskeva S., Hadjichristidis N., Thomas E.L. (2002). Synthesis and Microphase Separation of Linear Triblock Terpolymers of Polystyrene, High 1,4-Polybutadiene, and High 3,4-Polyisoprene. Macromolecules.

[52] Altintas O., Demirel A.L., Hizal G., Tunka U. (2008). Dendrimer-like miktoarm star terpolymers: A_3_-(B-C)_3_ via click reaction strategy. J. Polym. Sci. A Polym. Chem..

[53] Matsuo A., Watanabe T., Hirao A. (2004). Synthesis of Well-Defined Dendrimer-like Branched Polymers and Block Copolymer by the Iterative Approach Involving Coupling Reaction of Living Anionic Polymer and Functionalization. Macromolecules.

[54] Hirao A., Matsuo A., Watanabe T. (2005). Precise Synthesis of Dendrimer-like Star-Branched Poly(methyl methacrylate)s up to Seventh Generation by an Iterative Divergent Approach Involving Coupling and Transformation Reactions. Macromolecules.

[55] Lin B., Zhang H., Tang P., Qiu F., Yanga Y. (2011). Self-assembly of ABC dendrimer by real-space self-consistent mean field theory in a two-dimensional space. Soft Matter.

[56] van Hest J.C.M., Baars M.W.P.L., Elissen-Román C., van Genderen M.H.P., Meijer E.W. (1995). Acid-Functionalized Amphiphiles Derived from Polystyrene-Poly(propylene imine) Dendrimers, with a pH-Dependent Aggregation. Macromolecules.

[57] van Hest J.C.M., Delnoye D.A.P., Baars M.W.P.L., Elissen-Román C., van Genderen M.H.P., Meijer E.W. (1996). Polystyrene*-*PoIy(propylene imine) Dendrimers: Synthesis, Characterization, and Association Behavior of a New Class of Amphiphiles. Chem. Eur. J..

[58] Román C., Fischer H.R., Meijer E.W. (1999). Microphase Separation of Diblock Copolymers Consisting of Polystyrene and Acid-Functionalized Poly(propylene imine) Dendrimers. Macromolecules.

[59] Mackay M.E., Hong Y., Jeong M., Tande B.M., Wagner N.J., Hong S., Gido S.P., Vestberg R., Hawker C.J. (2002). Microphase Separation of Hybrid Dendron-Linear Diblock Copolymers into Ordered Structures. Macromolecules.

[60] Pochan D.J., Pakstis L., Huang E., Hawker C., Vestberg R., Pople J. (2002). Architectural Disparity Effects in the Morphology of Dendrimer-Linear Coil Diblock Copolymers. Macromolecules.

[61] Johnson M.A., Iyer J., Hammond P.T. (2004). Microphase Segregation of PEO-PAMAM Linear-Dendritic Diblock Copolymers. Macromolecules.

[62] Cho B.-K., Jain A., Nieberle J., Mahajan S., Wiesner U., Gruner S.M., Türk S., Räder H.J. (2004). Synthesis and Self-Assembly of Amphiphilic Dendrimers Based Aliphatic Polyether-Type Dendritic Cores. Macromolecules.

[63] Chung Y.-W., Lee B.-I., Kim H.-Y., Wiesner U., Cho B.-K. (2007). Influence of Crystalline Peripheral Chain Length on the Solid-State Assemblies of Amphiphilic Dendrons. J. Polym. Sci. A Polym. Chem..

[64] Cho B.-K., Kim S.-H., Lee E. (2010). Salt-induced microphase separation of amorphous dendritic poly(ethylene oxide)-block-linear polystyrene copolymers. J. Polym. Sci. A Polym. Chem..

[65] Moschovas D., Manesi G.-M., Karydis-Messinis A., Zapsas G., Ntetsikas K., Zafeiropoulos N.E., Piryazev A., Thomas E.L., Hadjichristidis N., Ivanov D.A. (2020). Alternating Gyroid Network Structure in an ABC Miktoarm Terpolymer Comprised of Polystyrene and Two Polydienes. Nanomaterials.

[66] Mark J.E. (2007). Physical Properties of Polymers Handbook, Part III: Thermodynamic Properties.

[67] Zapsas G., Moschovas D., Ntetsikas K., Rangou S., Lee J.-H., Thomas E.L., Zafeiropoulos N.E., Avgeropoulos A. (2015). Immiscible Polydiene Blocks in Linear Copolymer and Terpolymer Sequences. J. Polym. Sci. B Polym. Phys..

[68] Miller-Chou B.A., Koenig J.L. (2003). A review of polymer dissolution. Prog. Polym. Sci..

[69] Zhu Y.Q., Burgaz E., Gido S.P., Staudinger U., Weidisch R., Uhrig D., Mays J.W. (2006). Morphology and Tensile Properties of Multigraft Copolymers with Regularly Spaced Tri-, Tetra-, and Hexafunctional Junction Points. Macromolecules.

[70] Iatrou H., Hadjichristidis N. (1993). Morphology and Miscibility of Miktoarm Styrene-Diene Copolymers and Terpolymers. Macromolecules.

[71] Neumann C., Abetz V., Stadler R. (1998). Phase behavior of ABC-triblock copolymers with two inherently miscible blocks. Colloid Polym. Sci..

[72] Cohen R.E., Wilfong D.E. (1982). Properties of Block Copolymers and Homopolymer Blends Comprised of 1,2-Polybutadiene and 1,4-Polybutadiene. Macromolecules.

[73] Dair J.D., Avgeropoulos A., Hadjichristidis N., Thomas E.L. (2000). Mechanical properties of the double gyroid phase in oriented thermoplastic elastomers. J. Mater Sci..

[74] Mogi Y., Nomura M., Kotsuji H., Ohnishi K., Matsushita Y., Noda I. (1994). Superlattice Structures in Morphologies of the ABC Triblock Copolymers. Macromolecules.

[75] Brinkmann S., Stadler R., Thomas E.L. (1998). New Structural Motif in Hexagonally Ordered Cylindrical Ternary (ABC) Block Copolymer Microdomains. Macromolecules.

[76] Matsen M.W., Gardiner J.M. (2000). Anomalous domain spacing difference between AB diblock and homologous A_2_B_2_ starblock copolymers. J. Chem. Phys..

[77] Hayashida K., Dotera T., Takano A., Matsushita Y. (2007). Polymeric Quasicrystal: Mesoscopic Quasicrystalline Tiling in ABC Star Polymers. Phys. Rev. Lett..

