# Supplementary material for: Dendrons and Dendritic Terpolymers: Synthesis, Characterization and Self-Assembly Comparison
Source: Molecules. 2020 Dec 19;25(24):6030. doi: 10.3390/molecules25246030 (PMC7766238; doi:10.3390/molecules25246030)
Supplement: Supplementary file 1 [file molecules-25-06030-s001.pdf]

Dendrons and Dendritic Terpolymers: Synthesis, Characterization and Self-Assembly Comparison

Sofia Rangou[1,2], Dimitrios Moschovas[1,3], Ioannis Moutsios[1], Gkreti-Maria Manesi[1], Konstantina Tsitoni[1], Polina V. Bovsunovskaya[3,4], Dimitri A. Ivanov[3,4,5], Edwin L. Thomas[6], Apostolos Avgeropoulos[1,3,*]

[1] Department of Materials Science Engineering, University of Ioannina, University Campus-Dourouti, 45110 Ioannina, Greece; dmoschov@uoi.gr (D.M.); imoutsios@uoi.gr (I.M.); gretimanesi@uoi.gr (G.-M.M.); k.tsitoni@uoi.gr (K.T.)

[2] Helmholtz-Zentrum Geesthacht, Institut of Polymer Research, Max-Plank-Str. 1, 21502, Geesthacht, Germany; sofia.rangou@hzg.de (S.R.)

[3] Faculty of Chemistry, Lomonosov Moscow State University (MSU), GSP-1, 1-3 Leninskiye Gory, 119991 Moscow, Russia; bovspolina@yandex.ru (P.V.B.); dimitri.ivanov@uha.fr (D.A.I.)

[4] Institute of Problems of Chemical Physics, Russian Academy of Sciences, Chernogolovka, Moscow region, 142432, Russian Federation

[5] Institut de Sciences des Matériaux de Mulhouse – IS2M, CNRS UMR7361, 15 Jean Starcky, Mulhouse 68057, France

[6] Dept of Materials Science and Engineeering, Texas A&M University, College Station, TX 77843-3003, USA; elt@exchange.tamu.edu (E.L.T.)

* Correspondence: aavger@uoi.gr; Tel.: +3026-5100-9001

Supporting Information

The following data are given in the Supporting Information:

A). Size Exclusion Chromatography (SEC) Results

B). Proton Nuclear Magnetic Resonance Spectroscopy (^{1}H-NMR) Results

C). Differential Scanning Calorimetry (DSC) Results

A). Size Exclusion Chromatography (SEC) Results

In the following SEC chromatographs the initial blocks, the intermediate products, the miktoarm dendrons, the unfractionated dendritic and the final fractionated dendritic terpolymers are presented (Figures S1-S4). It is straightforward that, the final complex architecture terpolymers exhibit increased molecular and compositional homogeneity, since the dispersity index (Đ) is well below 1.1 for all cases. For all samples, in order to receive a purified final product, fractionation of the unpurified samples was employed using a toluene (solvent)/ methanol (non-solvent) system.

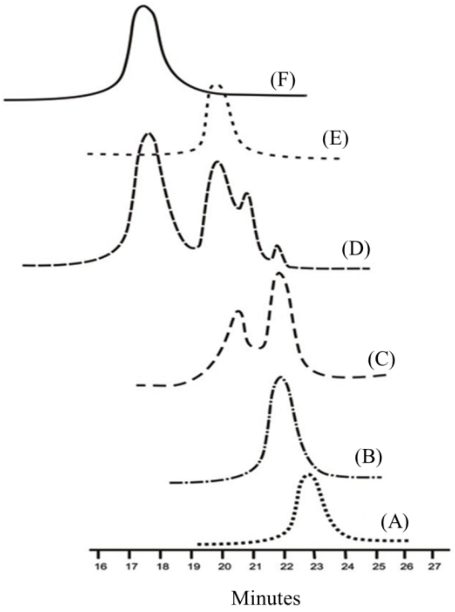

Figure S1. SEC chromatographs of: (A) the initial homopolymer PS, (B) the initial homopolymer PI, (C) synthesized CDMSS macro-initiator during linking reaction process, (D) final unfractionated dendritic terpolymer, (E) fractionated miktoarm dendron of (PS)(PI)(**PB**$^{-}$) type and (F) fractionated symmetric dendritic terpolymer of $[(PS)(PI)(PB)]_3$-B_3 core type.

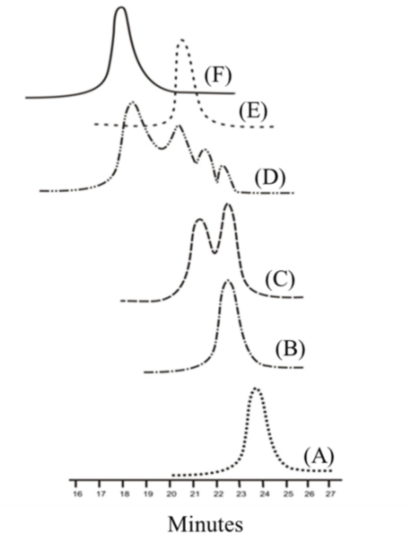

Figure S2. SEC chromatographs of: (A) the initial homopolymer PS, (B) the initial homopolymer PI, (C) synthesized CDMSS macro-initiator during linking reaction process, (D) unfractionated symmetric dendritic terpolymer, (E) fractionated miktoarm dendron of (PS)(PI)(**PB**$^{-}$) type and (F) fractionated symmetric dendritic terpolymer of [(PS)(PI)(PB)]$_4$-B$_4$ core type.

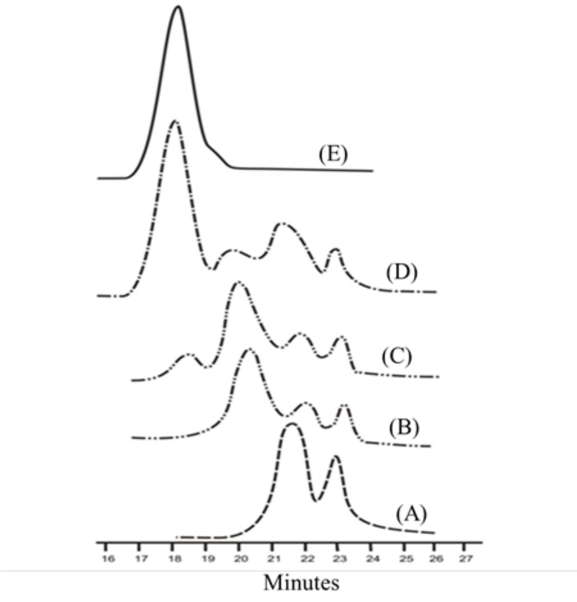

Figure S3. SEC chromatographs of: (A) miktoarm dendron of the (PB)(PS)(**PI**$^{-}$) type, (B) miktoarm dendron of the (PB)(PI)(**PS**$^{-}$) type, (C) living miktoarm dendron of (PB)(PI)(**PS**$^{-}$) type after coupling reaction with the CH_3SiCl_3, (D) unfractionated asymmetric dendritic terpolymer, (E) fractionated asymmetric dendritic terpolymer of [(PB)(PI)(PS$_c$)]-[(PS)(PB)(PI$_c$)]$_2$ type.

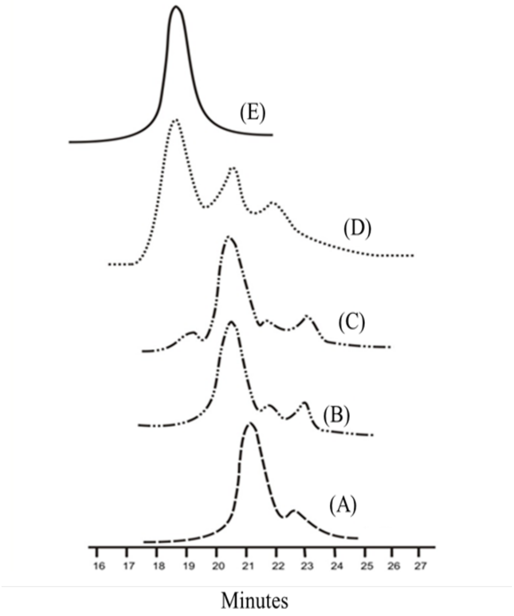

Figure S4. SEC chromatographs of: (A) miktoarm dendron of the (PB)(PS)(**PI**$^-$) type, (B) miktoarm dendron of the (PB)(PI)(**PS**$^-$) type, (C) living miktoarm dendron of (PB)(PS)(**PI**$^-$) type after coupling reaction with the $SiCl_4$, (D) unfractionated asymmetric dendritic terpolymer, (E) fractionated asymmetric dendritic terpolymer of [(PB)(PI)(PS$_c$)]-[(PS)(PB)(PI$_c$)]$_3$ type.

B). Proton Nuclear Magnetic Resonance Spectroscopy (^{1}H-NMR) Results

In Table S1 the type and number of protons per monomeric unit of each block, the geometric isomerism of the PI and PB blocks, as well as the corresponding chemical shifts, are presented in order to clarify the data taken from the ^{1}H-NMR spectra of the dendritic terpolymers. Through ^{1}H-NMR, as shown in Figure S5-S8, the successful synthesis was verified, given that chemical shifts corresponding to specific protons of the monomeric units of PS, PB and PI blocks are observed and through appropriate peak integration the segment composition was also calculated (Table S2).

Table S1. The type and number of protons per monomeric unit of each block as well as the chemical shifts, are presented in order to comprehend the ^{1}H-NMR spectra of the dendritic terpolymers.

Block	Geometric Isomerism	Type and Number of Protons	Chemical Shift (ppm)
PS	-	Aromatic (5)	6.30-7.37
PI	1,2	Olefinic (1)<br>Olefinic (2)	5.82<br>5.00
	1,4	Olefinic (1)	5.12
	3,4	Olefinic (2)	4.70
PB	1,4	Olefinic (2)	5.31
	1,2	Olefinic (1)<br>Olefinic (2)	5.60<br>4.95

In Table S2 the percentage of different geometric isomerisms of each polydiene for the final materials are presented.

Table S2. Block volume fraction ratios, as well as the geometric isomerism percentage of each polydiene are presented for all dendrons and final dendritic terpolymers as calculated through ^{1}H-NMR spectra.

**Sample**	**PS**	**PI (v/v %)<br>(1,2) (1,4) (3,4) (wt%)**	**PB (v/v %)<br>(1,2) (1,4) (wt%)**
(PS)(PI)(**PB**-)	0.37	0.32<br>(2) (31) (67)	0.31<br>(26) (74)
[(PS)(PI)(PB)]$_3$-B$_3$ core	0.37	0.32<br>(2) (31) (67)	0.31<br>(26) (74)
(PS)(PI)(**PB**-)	0.34	0.38<br>(2) (31) (67)	0.28<br>(20) (80)
[(PS)(PI)(PB)]$_4$-B$_4$ core	0.34	0.38<br>(2) (31) (67)	0.28<br>(20) (80)
(PB)(PI)(**PS**-)	0.65	0.20<br>(2) (31) (67)	0.15<br>(8) (92)
[(PB)(PI)(PS$_c$)]-[(PS)(PB)(PI$_c$)]$_2$<br>(SI$_2$ core)	0.36	0.29<br>(2) (31) (67)	0.35<br>(8) (92)
(PS)(PB)(**PI**-)	0.20	0.53<br>(2) (31) (67)	0.27<br>(8) (92)
(PB)(PI)(**PS**-)	0.53	0.22<br>(2) (31) (67)	0.25<br>(8) (92)
[(PB)(PI)(PSc)]-<br>[(PS)(PB)(PIc)]$_3$ (SI$_3$ core)	0.35	0.40<br>(2) (31) (67)	0.25<br>(8) (92)

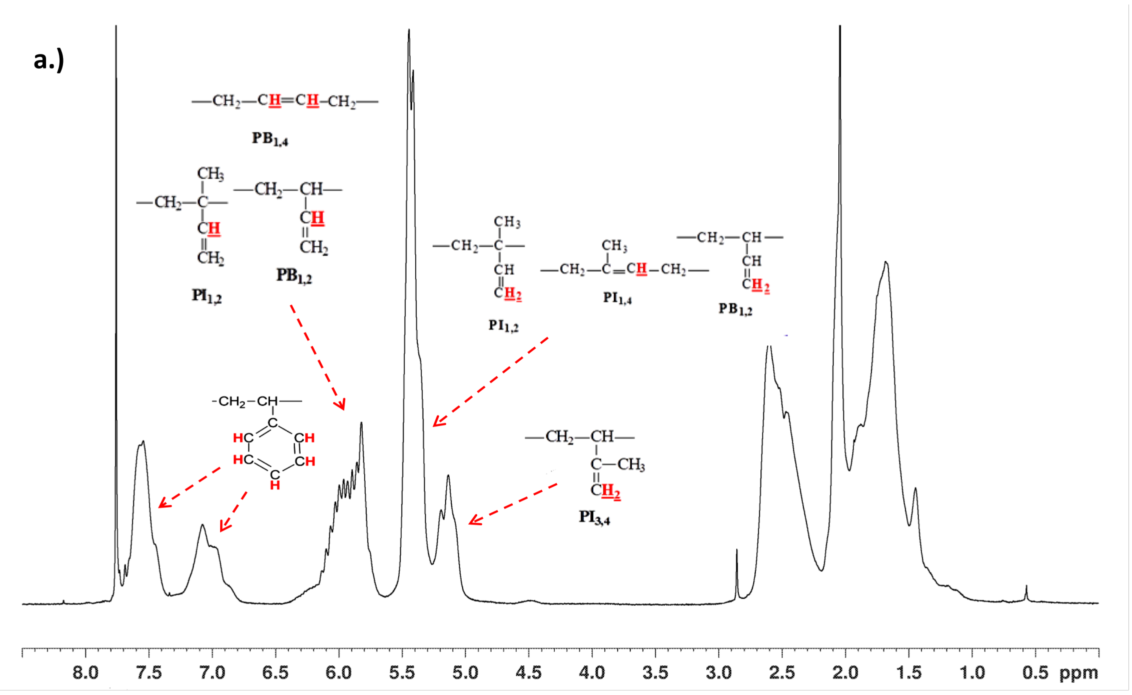

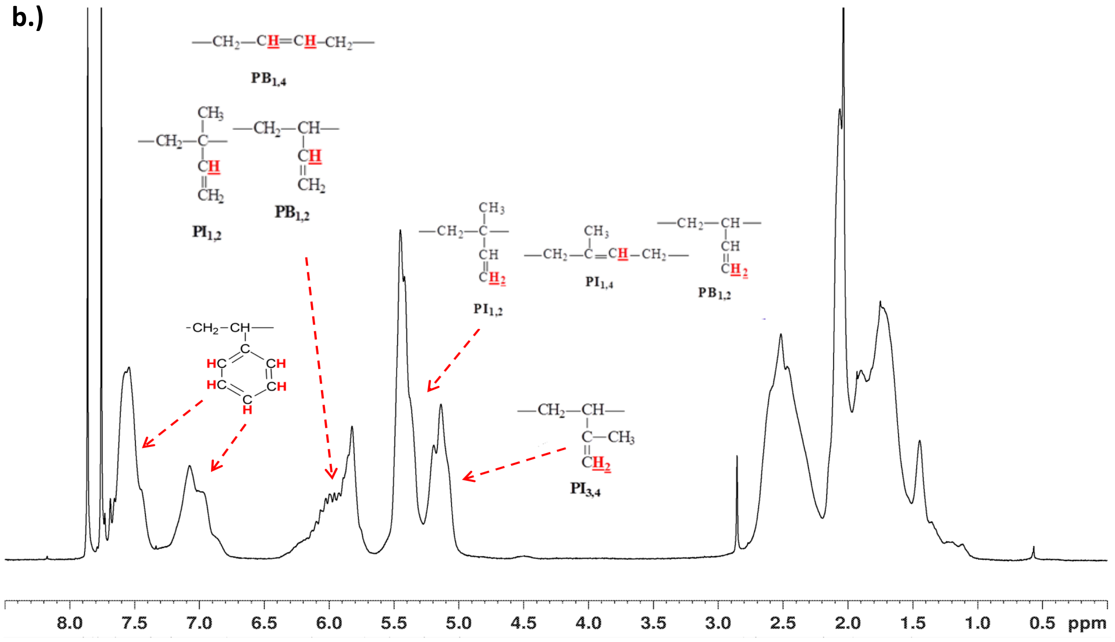

Figure S5. ^{1}H-NMR spectra of a.) the miktoarm dendron precursor of the [(PS)(PI)($\mathbf{PB}^{-}$)] type and b.) the symmetric dendritic terpolymer of the (PS)(PI)(PB)]$_3$-B$_3$ core type.

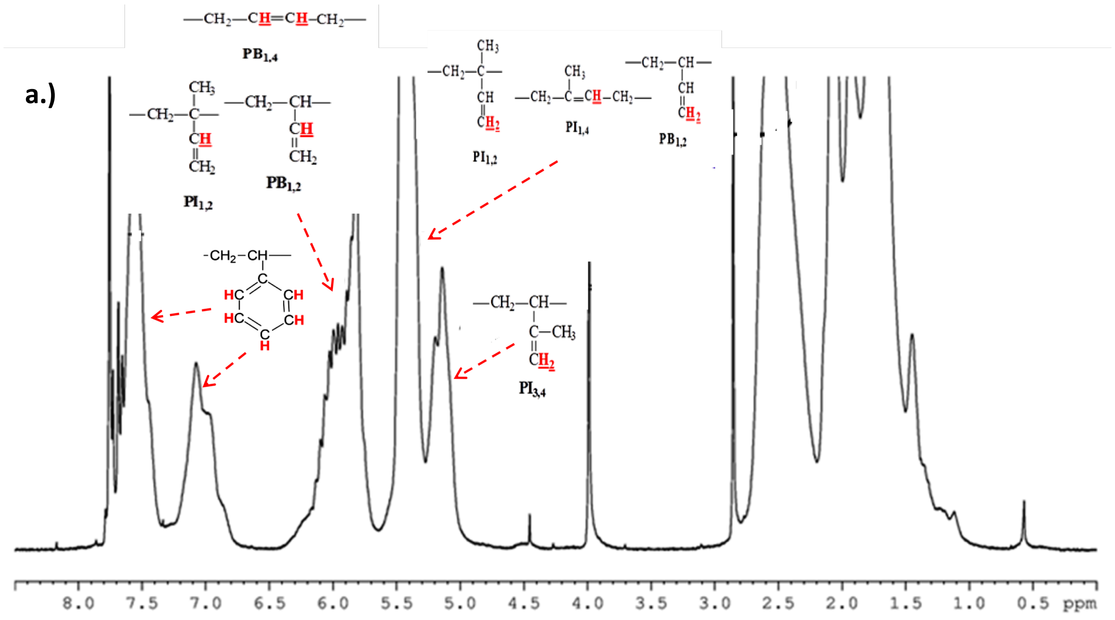

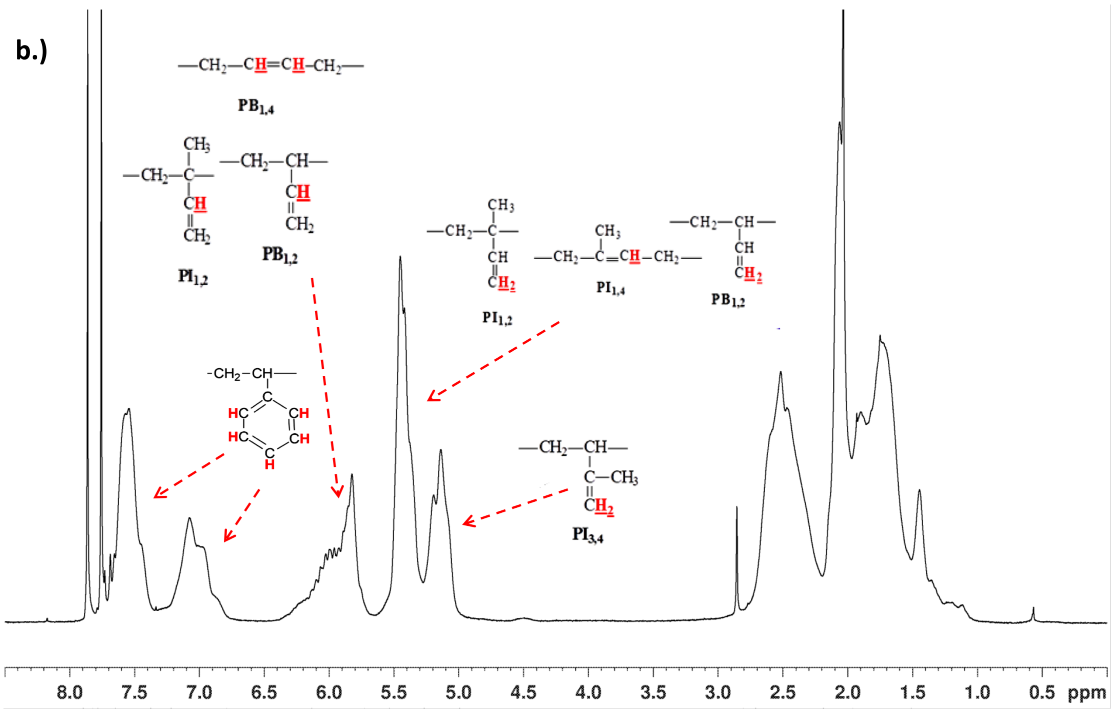

Figure S6. ^{1}H-NMR spectra of a.) the miktoarm dendron precursor of the [(PS)(PI)(**PB**$^{-}$)] type and b.) the symmetric dendritic terpolymer of the (PS)(PI)(PB)]$_4$-B$_4$ core type.

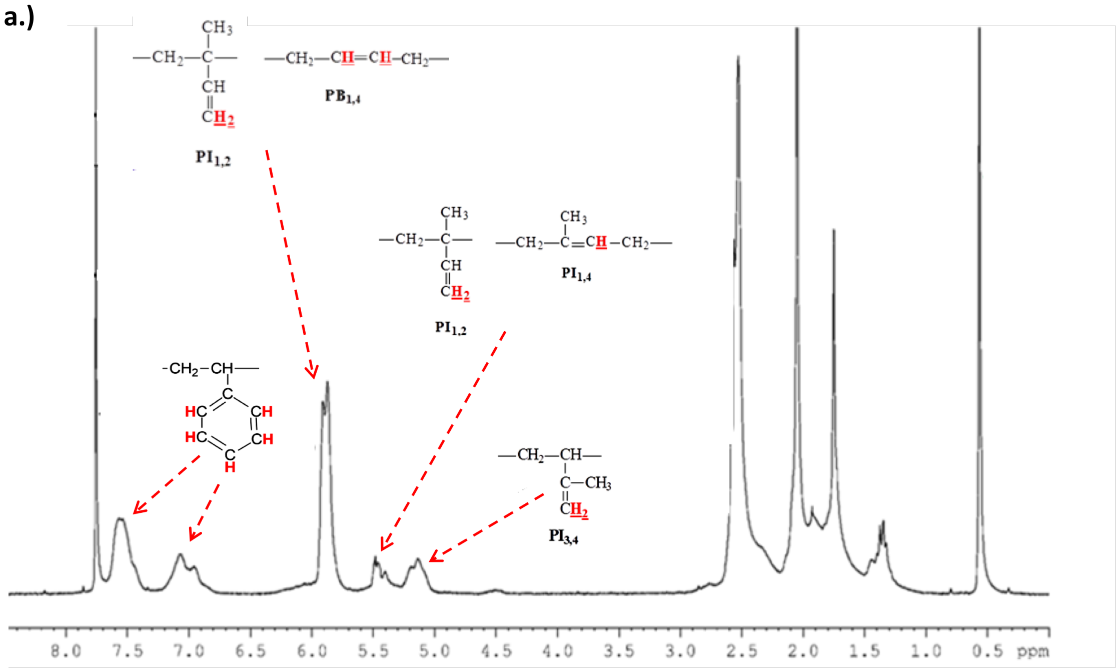

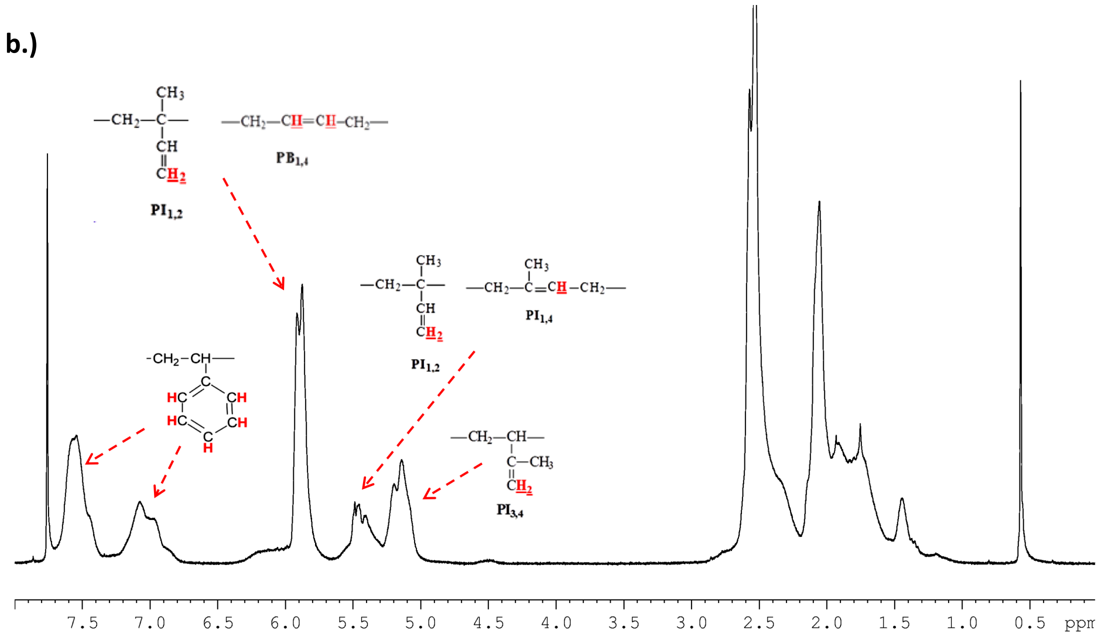

Figure S7. ^{1}H-NMR spectra of a.) the miktoarm dendron precursor of the (PB)(PI)(**PS**$^{-}$) type and b.) the asymmetric dendritic terpolymer of the [(PB)(PI)(PS$_c$)]-[(PS)(PB)(PI$_c$)]$_2$ type.

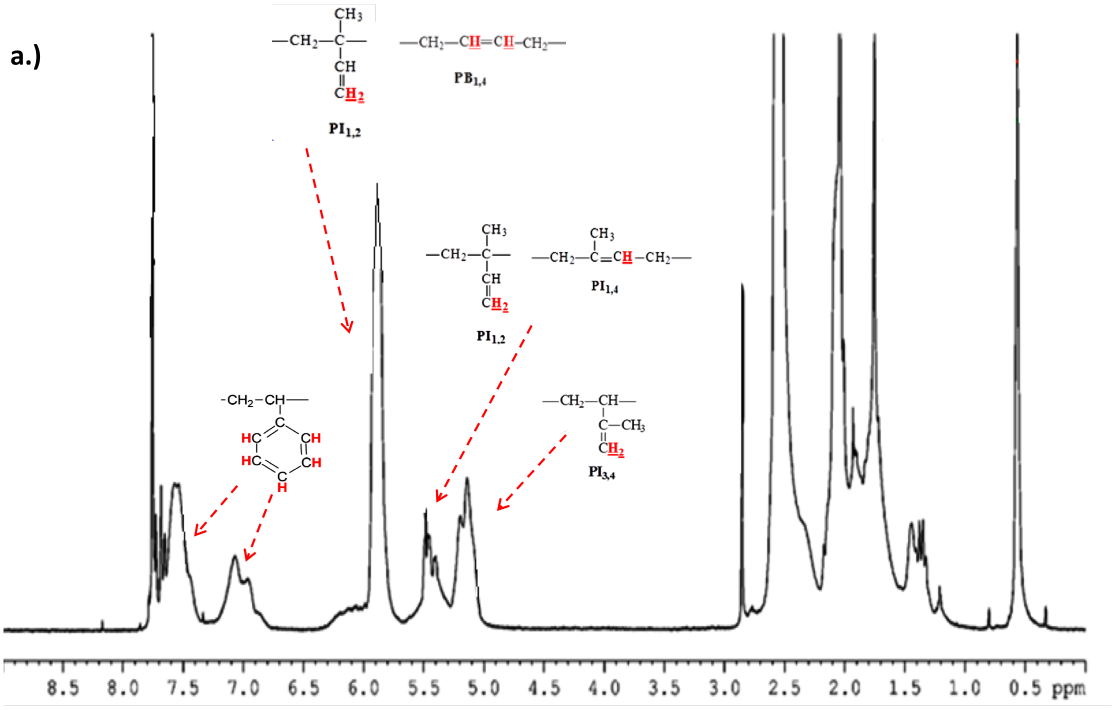

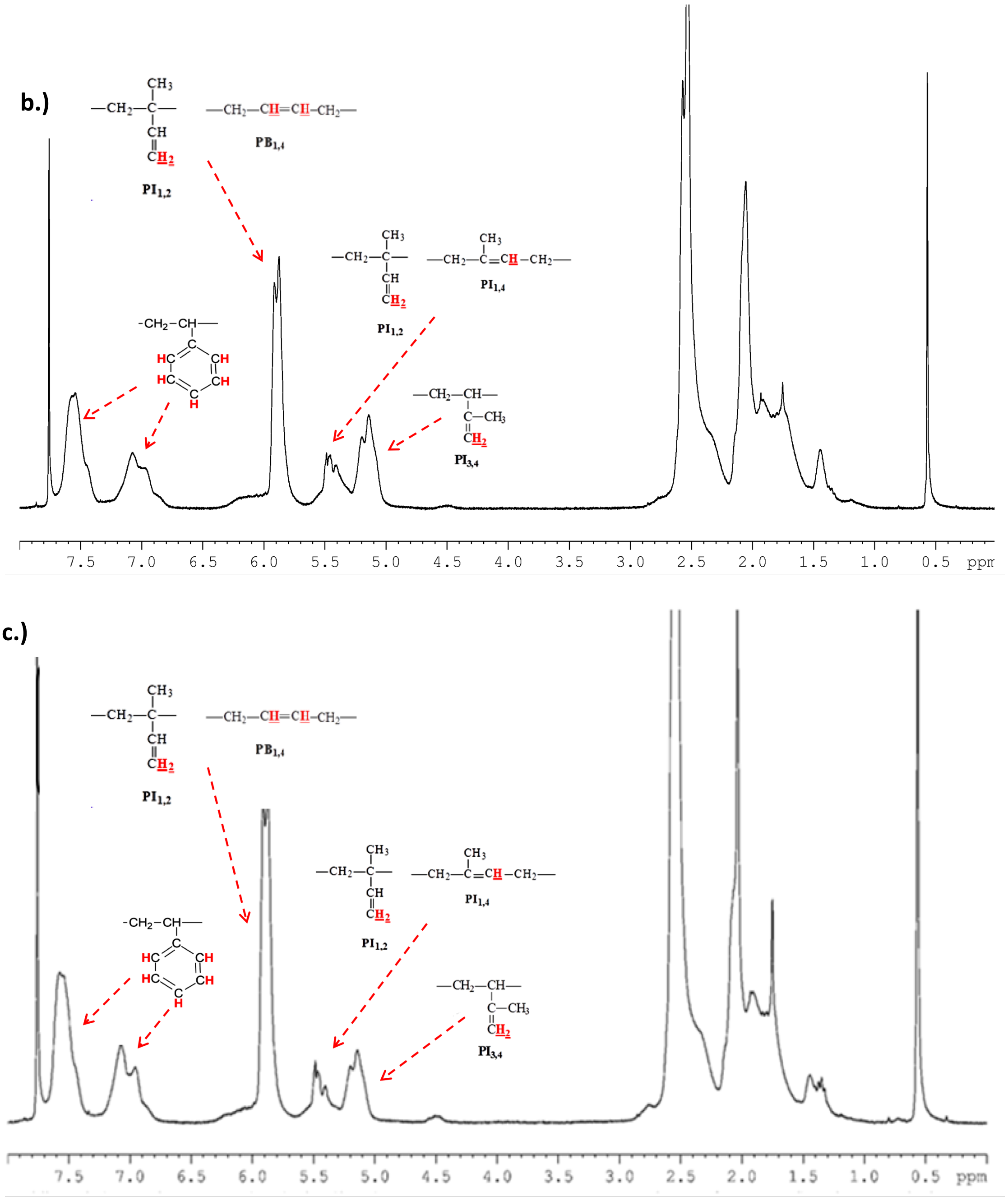

Figure S8. ^{1}H-NMR spectra of a.) the miktoarm dendron precursor of the (PB)(PI)(**PS**$^{-}$) type, b.) the miktoarm dendron precursor of the (PS)(PB)(**PI**$^{-}$) type and c.) the asymmetric dendritic terpolymer of [(PB)(PI)(PS$_c$)]-[(PS)(PB)(PI$_c$)]$_3$ type.

C). Differential Scanning Calorimetry (DSC) Results

In Figures S9-S13 the characteristic glass transition temperatures T_gs for the PS, PB and PI blocks are observed. It should be mentioned that both DSC measurements of the dendrons and the dendritic terpolymers are presented in the same thermograph for comparison reasons. In all cases the final dendritic sequences are depicted with solid green lines, while the dendron precursors are illustrated with dash green lines. In Table S3 the glass transition temperatures for the three different segments are given for all dendrons and final dendritic terpolymers.

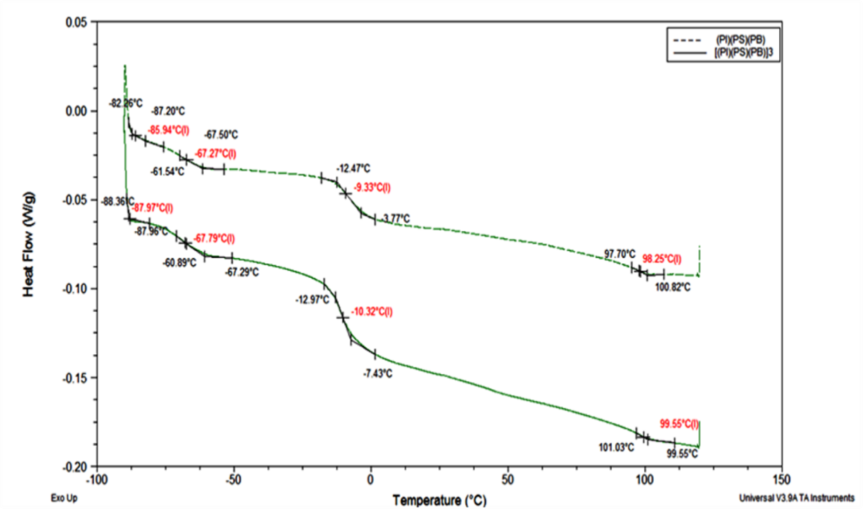

Figure S9. DSC thermograph of the (PS)(PI)(**PB**$^-$) miktoarm dendron precursor (dash line) and the symmetric [(PS)(PI)(PB)]$_3$- B$_3$ core dendritic terpolymer (solid line).

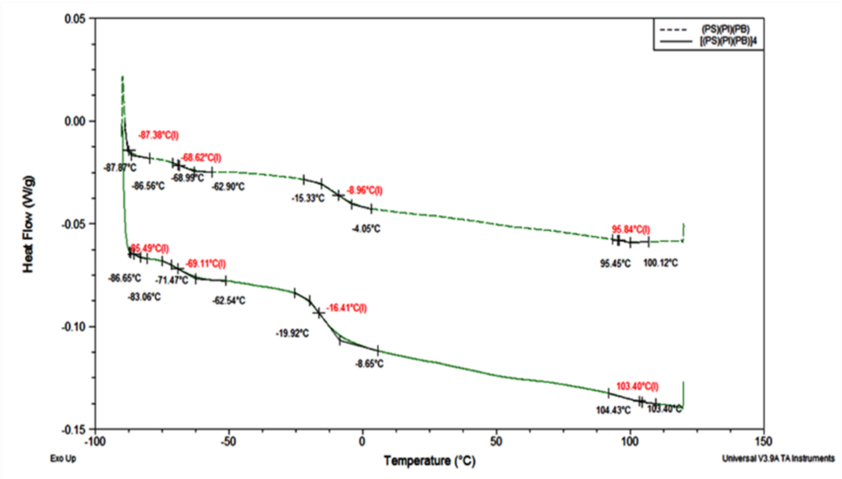

Figure S10. DSC thermograph of the (PS)(PI)(**PB**$^-$) miktoarm dendron precursor (dash line) and the symmetric [(PS)(PI)(PB)]$_4$- B$_4$ core dendritic terpolymer (solid line).

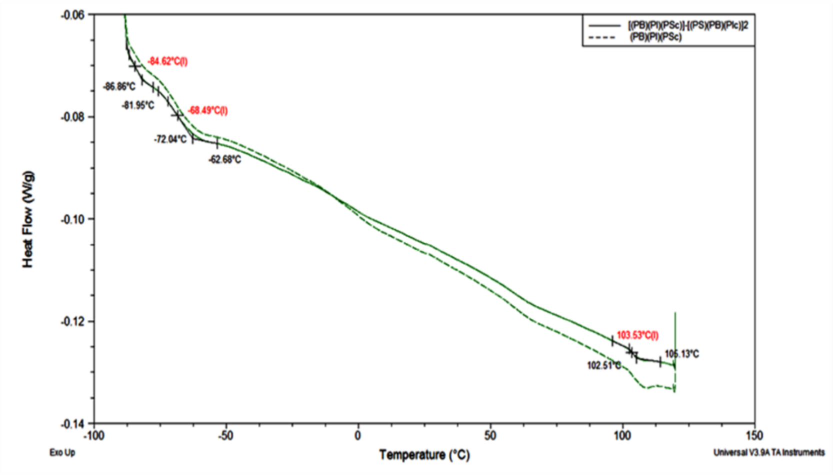

Figure S11. DSC thermograph of the (PB)(PI)(**PS**$^-$) miktoarm dendron precursor (dash line) and the asymmetric [(PB)(PI)(PS$_c$)]-[(PS)(PB)(PI$_c$)]$_2$ dendritic terpolymer (solid line).

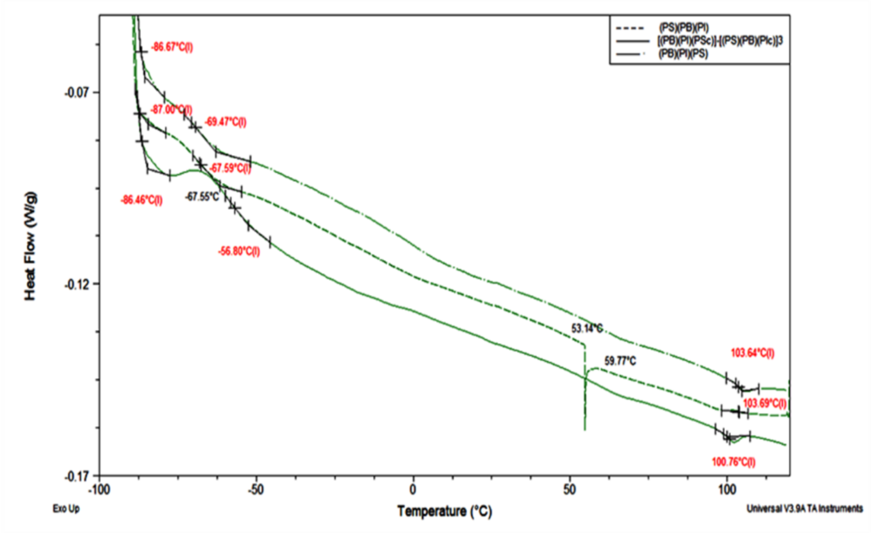

Figure S12. DSC thermograph of the (PS)(PB)(**PI**$^-$) miktoarm dendron precursor (dash line), (PB)(PI)(**PS**$^-$) miktoarm dendron precursor (dash-dot line) and the asymmetric [(PB)(PI)(PS$_c$)]-[(PS)(PB)(PI$_c$)]$_3$ dendritic terpolymer (solid line).

Table S3. Glass transition temperatures (T_g) of all synthesized materials.

**Sample**	T_g^{PS} (°C)	$T_g^{PB1,4}$ (°C)	$T_g^{PI3,4}$ (°C)	$T_g^{PB1,2}$ (°C)
(PS)(PI)(**PB**$^-$)	98	-85	-67	-9
[(PS)(PI)(PB)]$_3$-B$_3$ core	99	-88	-67	-10
(PS)(PI)(**PB**$^-$)	96	-87	-69	-9
[(PS)(PI)(PB)]$_4$-B$_4$ core	103	-86	-69	-16
(PB)(PI)(**PS**$^-$)	103	-85	-69	x
[(PB)(PI)(PS$_c$)]-[(PS)(PB)(PI$_c$)]$_2$ (SI$_2$ core)	103	-84	-68	x
(PS)(PB)(**PI**$^-$)	103	-87	-68	x
(PB)(PI)(**PS**$^-$)	103	-86	-69	x
[(PB)(PI)(PS$_c$)]-[(PS)(PB)(PI$_c$)]$_3$ (SI$_3$ core)	100	-87	-57	x
